# Choosing a camera and optimizing system parameters for speckle contrast optical spectroscopy

**DOI:** 10.1038/s41598-024-62106-y

**Published:** 2024-05-24

**Authors:** Tom Y. Cheng, Byungchan Kim, Bernhard B. Zimmermann, Mitchell B. Robinson, Marco Renna, Stefan A. Carp, Maria Angela Franceschini, David A. Boas, Xiaojun Cheng

**Affiliations:** 1 Department of Biomedical Engineering, Neurophotonics Center, Boston University, Boston, MA 02215 USA; 2 Department of Radiology, Athinoula A. Martinos Center for Biomedical Imaging, Massachusetts General Hospital, Harvard Medical School, Charlestown, MA 02129 USA; 3 Lincoln Laboratory, Massachusetts Institute of Technology, Lexington, MA 02421 USA

**Keywords:** Biomedical engineering, Blood flow

## Abstract

Speckle contrast optical spectroscopy (SCOS) is an emerging camera-based technique that can measure human cerebral blood flow (CBF) with high signal-to-noise ratio (SNR). At low photon flux levels typically encountered in human CBF measurements, camera noise and nonidealities could significantly impact SCOS measurement SNR and accuracy. Thus, a guide for characterizing, selecting, and optimizing a camera for SCOS measurements is crucial for the development of next-generation optical devices for monitoring human CBF and brain function. Here, we provide such a guide and illustrate it by evaluating three commercially available complementary metal–oxide–semiconductor cameras, considering a variety of factors including linearity, read noise, and quantization distortion. We show that some cameras that are well-suited for general intensity imaging could be challenged in accurately quantifying spatial contrast for SCOS. We then determine the optimal operating parameters for the preferred camera among the three and demonstrate measurement of human CBF with this selected low-cost camera. This work establishes a guideline for characterizing and selecting cameras as well as for determining optimal parameters for SCOS systems.

## Introduction

Cerebral blood flow (CBF) is a critical biomarker of brain health. Abnormal alterations in CBF can be related to serious neurological conditions such as ischemic stroke^1–3^, traumatic brain injury^4–6^, and Alzheimer’s disease^7–9^. Variations in CBF are also an indicator of brain activity due to neurovascular coupling^10–14^. Thus, an accessible and accurate method for monitoring CBF holds high potential to advance medicine and neuroscience. Speckle contrast optical spectroscopy (SCOS) is a technique that has been developed theoretically^15–19^ and experimentally^20–24^ in the past decade to measure CBF noninvasively. Recently, it has been demonstrated that by appropriately selecting measurement parameters, SCOS can achieve more than an order-of-magnitude improvement in signal-to-noise ratio (SNR) at reduced cost as compared to diffuse correlation spectroscopy (DCS), the current state-of-the-art optical technique for measuring human CBF^25,26^. In SCOS, the speckle patterns which are the interference patterns generated by coherent backscattered light from the tissue, are recorded. The spatial contrast of the speckle pattern, defined as the standard deviation divided by the mean of the speckle intensity, $K=\sigma \left(I\right)/\langle I\rangle$, is calculated and a blood flow index (BFi) can be quantified from the blood-flow-induced reduction of the spatial contrast within a certain camera exposure time. Complementary metal–oxide–semiconductor (CMOS) cameras are typically used to image dynamic speckle patterns due to their high frame rate and cost-effectiveness as compared to other camera technologies such as charge-coupled device (CCD) cameras^27,28^. A major challenge in SCOS is that, besides impacting SNR, CMOS camera noise and nonidealities can also impact the accuracy of SCOS measurements in the low-photon-flux regime relevant to human brain measurements. A noise correction procedure has been developed to improve SCOS measurement accuracy^25,26,29^, which requires precise characterization of camera properties including camera gain and pixel-wise dark offset and read noise. In the course of this work, we have found that the summary values provided in a camera datasheet are usually insufficient for SCOS noise correction and do not reveal camera nonidealities, such as second-order nonlinearity and quantization distortion, that can impact a camera’s suitability for SCOS. Thus, a guide for characterizing a camera, determining its suitability for SCOS measurements, and then obtaining the optimal operating parameters for a selected camera is needed to aid a broad set of users in developing optimized SCOS systems to measure human CBF and brain function.

The objective of this work is to establish a guideline for characterizing, selecting, and optimizing a camera for the application of measuring human CBF using SCOS. To demonstrate the guideline, we investigated a scenario where one would choose among three commercially available CMOS cameras (Hamamatsu Orca Fusion BT C15440-20UP scientific CMOS camera, denoted as HA; Basler a2A1920-160umPRO camera, denoted as BAa; Basler daA1280-54um board-level camera, denoted as BAd) considering factors including first-order and second-order linearity, read noise, quantization distortion, and cost. Among other findings, we observed that some cameras that are well-suited for imaging applications primarily concerned with accurately recovering intensity information may not recover as accurately the variance of intensity which is used in SCOS. We then use our recently developed noise model^25^ to illustrate how to find the set of parameters—including the speckle-to-pixel size ratio (s/p), camera exposure time ($T_{\exp }$), and laser pulsing factor (PF), defined as the inverse of the duty cycle of a pulsed laser—for optimal shot-noise-limited SCOS measurement SNR using our selected, relatively low-cost BAa camera. We demonstrate that, with optimal selection of parameters for the BAa camera, we can measure pulsation-resolved human CBF at a lower cost than done before with our previous SCOS system^26^. This work lays a foundation for designing future affordable SCOS systems to measure human CBF.

## Methods

### Characterization of the camera’s gain, dark offset, and read noise

**Figure 1:**
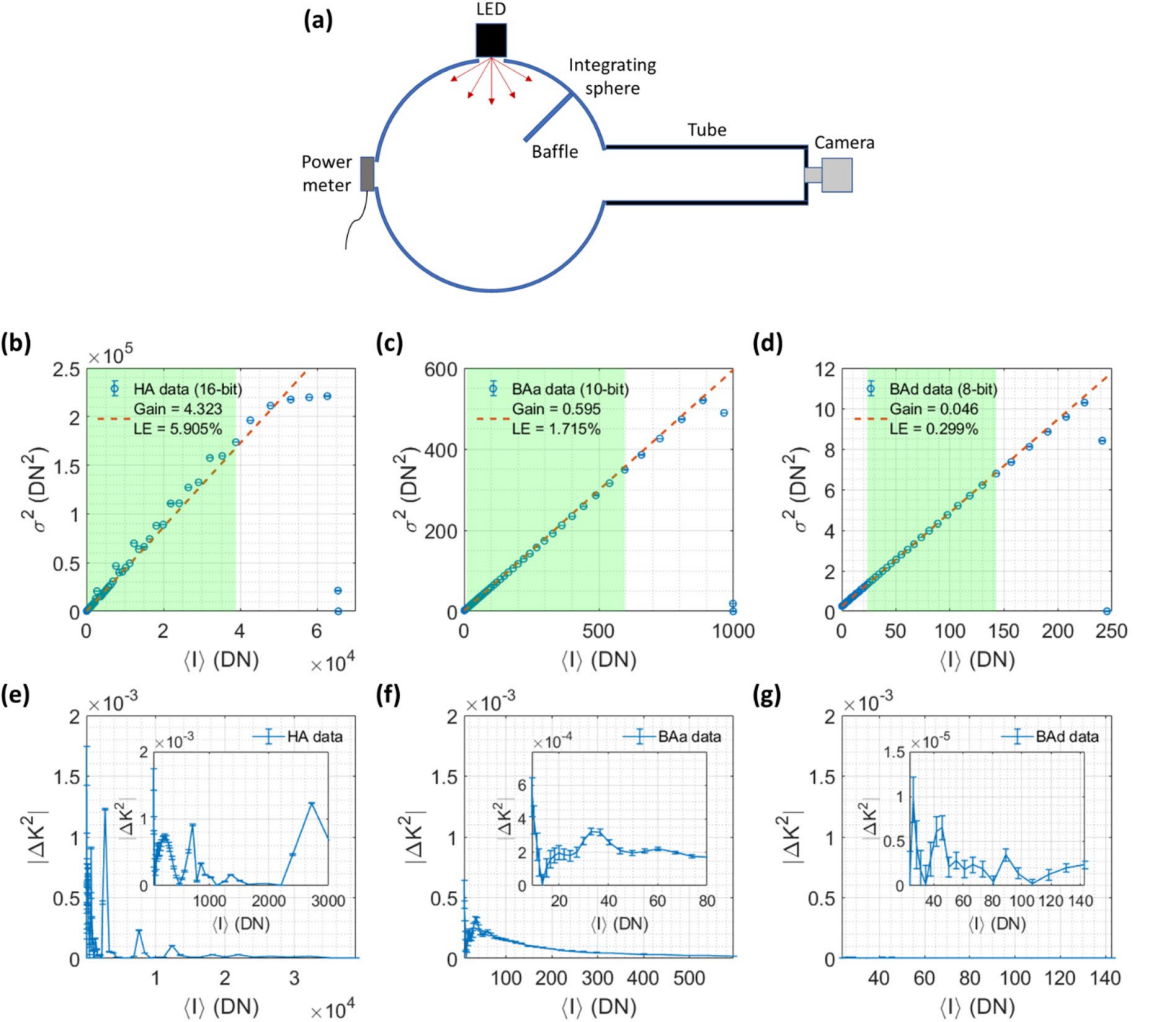
Characterization of camera gain and nonlinearity-induced K^2^ error for three CMOS cameras. (**a**) Schematic of the setup for measuring the cameras’ photon transfer curves. (**b**–**d**) Estimation of camera gain and linearity error (LE) for each camera’s photon transfer curve. Here ${\sigma }^{2}$ is the variance of the intensity and $\langle I\rangle$ is the average intensity across all the pixels, in units of the camera’s digital number (DN) output. Blue circles are the experimental data and red dashed lines are the linear regression lines. Error bars represent the temporal standard deviation of the variance of each difference image obtained. The linear regression is performed on data within a limited $\langle I\rangle$ range as indicated by the shaded region, where the shot noise is greater than twice the read noise and $\langle I\rangle$ is less than 70% of the camera’s saturation capacity. The estimated camera gain is the slope of the regression line. The LE is calculated as the mean magnitude of the relative deviation of the measured variance from the regression line within the fitting range. (**e**–**g**) Estimation of the absolute nonlinearity-induced error in ${K}^{2}$, $|\Delta {K}^{2}|$, within the shaded region indicated in (**b**–**d**). Error bars represent the temporal standard deviation of the variance of each difference image divided by ${\langle I\rangle }^{2}$. HA: Hamamatsu Orca Fusion BT C15440-20UP; BAa: Basler a2A1920-160umPRO; BAd: Basler daA1280-54um.

The first step after receiving a camera is to characterize the camera gain $g$, per-pixel dark offset ${\langle I\rangle }_{{\rm dark}}\left(x,y\right)$, and per-pixel read noise ${\sigma }_{{r}}\left(x,y\right)$ which are needed for the noise correction procedure described in a later section. These steps will also help to determine the suitability of a given camera for SCOS measurements. The schematic of the characterization setup is shown in Fig. 1a. We used an integrating sphere as a uniform illumination source and placed the camera 40 cm away from the integrating sphere’s exit port which has a diameter of 5 cm. At this distance, the irradiance uniformity is predicted to be over 99% for a sensor with diagonal dimension equal to the diameter of the port, and higher for smaller sensors^30^. We mounted a temperature-stable incoherent light source (Thorlabs M730L5 LED) to one of the 2 input ports, and a photodiode power meter (Thorlabs S120C) to the other input port. A baffle is installed inside the integrating sphere between the LED port and exit port to ensure that there is no direct light path between the source and the exit port. A direct path between the source and the portion of the inner sphere wall in direct view of the detector was also eliminated by increasing the distance between the light source and the input port. The optical power meter monitored the illumination intensity as we swept the LED current to cover the camera’s intensity range. Note that monitoring the optical power is not necessary for measuring the camera’s gain $g$. We only did so to measure the camera’s first-order and second-order linearities (Fig. S1). To minimize introduction of ambient light and environmental reflections, we enclosed the light path between the integrating sphere’s exit port and the camera using a series of matte-black coated tubes (Thorlabs SM2). The camera was mounted at the end of the tube using a C-mount to SM2 adapter (Thorlabs SM2A31). We obtained the per-pixel ${\langle I\rangle }_{{\rm dark}}\left(x,y\right)$ and ${\sigma }_{{r}}\left(x,y\right)$ by collecting 100 dark images with no incident light on the camera, and then calculating the temporal mean and variance of each pixel. The camera exposure time expected to be used in the intended SCOS measurement should be used during camera characterization to account for any dark-current-induced offset and noise.

We obtained the photon transfer curve by illuminating the camera at different intensities using the LED source and obtaining 100 frames at each intensity, after which we subtracted the ${\langle I\rangle }_{{\rm dark}}\left(x,y\right)$ image from each frame. We used the statistics feature in the Thorlabs Optical Power Monitor software to take a 10-s moving average of the photodiode power meter reading and used the result from the moving average as the optical power value at each data point. We collected at least 50 data points across the camera’s intensity range. We collected the data points at logarithmically spaced intensity values up until 70% of the camera’s saturation intensity, ${\langle I\rangle }_{{\rm sat}}$, defined as the $\langle I\rangle$ at which the intensity histogram from an image starts to become clipped at the camera’s maximum intensity value. This was done in the interest of obtaining more points at the lower intensity values relevant to human CBF measurements while limiting the total number of measurements required. Beyond 70% of the camera’s ${\langle I\rangle }_{{\rm sat}}$, we collected approximately evenly spaced data points to show the camera’s behavior around saturation.

To minimize bias in the calculated mean variance from illumination nonuniformity and instability, we computed difference images from the frames and calculated the mean spatial variance of the difference images.

For estimation of camera gain from the photon transfer curve, we used a weighted least-squares linear fit with weights $1/{\langle I\rangle }^{2}$ calculated using data points within our chosen operating intensity range. For all three cameras, we chose the lower operating limit to be the $\langle I\rangle$ at which shot noise is equal to twice the camera’s read noise, and we chose the upper operating limit to be 70% of the camera’s ${\langle I\rangle }_{{\rm sat}}$. We chose a weighted linear fit to reduce fitting error and improve estimation of camera gain at lower intensities. Generally, the user should choose a fitting strategy that most accurately estimates the camera gain within the intensity region that the user expects to utilize.

Note that a camera’s performance parameters are generally influenced by the camera’s configuration settings. We used the following settings for the three cameras. HA: 16-bit, fast scan mode; BAa: 10-bit depth, 16 dB analog gain; BAd: 8-bit depth, 0 dB digital gain (no analog gain available).

### Characterization of camera dark offset and read noise variability

Twenty-five measurements of pixel-averaged dark offset ${\langle I\rangle }_{{\rm dark}}$ and read noise variance ${\sigma }_{{r}}^{2}$ were performed at a rate of one measurement every 10 min, with the camera sensor covered to ensure no light was incident on the sensor. Between measurements, the camera continued to acquire images but did not save them. The camera was powered on but not acquiring images for 3 h prior to the first measurement, to allow for measuring the effect of camera warm-up on the read noise variability. The ${\sigma }_{{r}}^{2}$ values were calculated using difference images in the same way as described in the previous section. The per-pixel ${\langle I\rangle }_{{\rm dark}}\left(x,y\right)$ and ${\sigma }_{{r}}^{2}\left(x,y\right)$ were calculated from the pixel-wise temporal mean and variance across 1000 frames.

### Model for quantization distortion and associated K^2^ error

For the simulation of quantization distortion, we used 400 evenly spaced true mean values ${\langle I\rangle }_{{\rm true}}$ within the quantization interval between −0.5 DN and 0.5 DN, and 100 true variance values ${\sigma }_{{\rm true}}^{2}$ between 0 DN^2^ and 1 DN^2^. We created a normal distribution for each possible combination of ${\langle I\rangle }_{{\rm true}}$ and ${\sigma }_{{\rm true}}^{2}$ values, with the assumption that the temporal distribution of read noise and shot noise can be approximated well with a normal distribution. We then quantized the values in each normal distribution and estimated the mean intensity $\langle I\rangle$ and variance ${\sigma }^{2}$ using the quantized values. We calculated the estimation error in the $\langle I\rangle$ and ${\sigma }^{2}$ for each combination of ${\langle I\rangle }_{{\rm true}}$ and ${\sigma }_{{\rm true}}^{2}$ values and found the maximum estimation error across all ${\langle I\rangle }_{{\rm true}}$ values at each ${\sigma }_{{\rm true}}^{2}$ value.

To calculate the associated ${K}^{2}$ percentage error, we used the following expression based on the propagation of error formula for $f=A/B$,

$$\frac{\Delta \left({K}^{2}\right)}{{K}^{2}}=\sqrt{{\left(\frac{\Delta \left({\sigma }^{2}\right)}{{\sigma }^{2}}\right)}^{2}+{\left(\frac{\Delta \left({\langle I\rangle }^{2}\right)}{{\langle I\rangle }^{2}}\right)}^{2}-\frac{{\rm Cov}\left({\sigma }^{2},{\langle I\rangle }^{2}\right)}{{\sigma }^{2}{\langle I\rangle }^{2}}}$$

where $\Delta \left(\dots \right)$ is the deviation of the estimated value from the true value. For a conservative estimate of the ${K}^{2}$ error, we assumed that ${\sigma }^{2}$ and ${\langle I\rangle }^{2}$ were independent, meaning that ${\rm Cov}\left({\sigma }^{2},{\langle I\rangle }^{2}\right)=0$. We found the maximum ${K}^{2}$ error across all ${\langle I\rangle }_{{\rm true}}$ values at each ${\sigma }_{{\rm true}}^{2}$ value. Note that using a quantization interval farther away from zero will result in a slightly lower ${K}^{2}$ error at a given ${\sigma }_{{\rm true}}^{2}$ value, due to a decrease in the $\frac{\Delta \left({\langle I\rangle }^{2}\right)}{{\langle I\rangle }^{2}}$ term in the above formula.

### Noise correction procedure for SCOS measurements

The aim of noise correction for SCOS is to correct for the biases in the speckle contrast contributed by shot noise, read noise, quantization noise, and spatial nonuniformity in illumination. Shot noise arises from the random variation in the number of incident photons on each pixel. Read noise is the aggregate random variation in the camera’s intensity reading arising from the processes of converting photoelectrons to a voltage, amplifying the voltage, and digitizing the voltage. The act of estimating the variance in intensity from quantized intensity values such as the camera’s digital output introduces an additional bias of 1/12 in the measured variance according to quantization theory^31,32^. The added spatial variance from spatially nonuniform illumination on the sensor must also be subtracted. We recently developed an experimentally validated noise correction procedure^26^ for SCOS which corrects for the above noise sources and is summarized below.

We first subtract the mean dark image from the raw speckle images. We then select the region of interest (ROI) in the speckle image for the speckle contrast calculation, typically chosen to match the fiber output image. We calculate the raw speckle contrast squared ${K}_{{\rm raw}}^{2}=(\sigma \left(I\right)/\langle I\rangle {)}^{2}$ for each 7 × 7 pixel window within the ROI, where the intensity $I$ is measured in digital numbers (DN) for all pixels. We use linear fitting to reduce the noise in each window’s mean intensity time course, as described previously^26^ and summarized below. We first obtain the average intensity time course ${I}_{{\rm all}}(t)$ across the ROI. We assume that the average intensity time course for each window, ${I}_{{w}}(t)$, is linearly related to ${I}_{{\rm all}}(t)$ and given by ${I}_{{w}}\left(t\right)={a\cdot I}_{{\rm all}}\left(t\right)+b$. The coefficients $a$ and $b$ are obtained from the fitting. The resulting fit, $\langle I(t)\rangle ={a\cdot I}_{{\rm all}}\left(t\right)+b$, is used in the denominator of ${K}_{{\rm raw}}$ for each window. After obtaining the raw contrast squared ${K}_{{\rm raw}}^{2}$, we subtract the speckle contrast bias terms arising from shot noise ${K}_{{s}}^{2}$, read noise ${K}_{{r}}^{2}$, spatial nonuniformity in illumination ${K}_{{\rm sp}}^{2},$ and quantization bias ${K}_{{q}}^{2}$ as follows,

$$K_{{\text{f}}}^{2} = K_{{{\text{raw}}}}^{2} - K_{{\text{s}}}^{2} - K_{{\text{r}}}^{2} - K_{{{\text{sp}}}}^{2} - K_{{\text{q}}}^{2} ,\;K_{{\text{s}}}^{2} = \frac{g}{\left\langle I \right\rangle },\;K_{{\text{r}}}^{2} = \frac{{\sigma_{{\text{r}}}^{2} }}{{\left\langle I \right\rangle^{2} }},\;K_{{{\text{sp}}}}^{2} = \frac{{\sigma_{{{\text{sp}}}}^{2} }}{{\left\langle I \right\rangle^{2} }},\;K_{{\text{q}}}^{2} = \frac{1/12}{{\left\langle I \right\rangle^{2} }},$$

where $g$ is the camera gain, ${\sigma }_{{r}}$ is the read noise of the camera, and ${\sigma }_{{sp}}^{2}$ is the spatial variance obtained from a temporal average of the speckle images. For ${K}_{{q}}^{2}$, the quantization-induced bias in variance is assumed to be 1/12^31,32^. We then obtain an intensity-weighted average of ${K}_{{f}}^{2}$, the “fundamental” blood-flow-induced contrast squared, across all the 7 × 7 windows within the ROI to obtain a single ${K}_{{f}}^{2}$ value for each camera frame.

### Analytical noise model for SCOS

The noise model for SCOS has been developed and experimentally validated previously^25^ and is summarized here. The noise in SCOS measurements $\sigma ({K}_{{\rm raw}}^{2})$ is the root-sum-square of the individual contributions from fundamental noise, shot noise, and read noise:

$$\sigma \left({K}_{{\rm raw}}^{2}\right)=\sqrt{{\sigma }^{2}\left({K}_{{f}}^{2}\right)+{\sigma }^{2}\left({K}_{{s}}^{2}\right)+{\sigma }^{2}\left({K}_{{r}}^{2}\right)}$$

Each noise term is calculated as follows,

$$\sigma \left( {K_{{\text{f}}}^{2} } \right) = K_{{\text{f}}}^{2} \sqrt {\frac{{2\left( {1 + 2K_{{\text{f}}}^{2} } \right)}}{{{\text{NIO}} \cdot N_{{{\text{fr}}}} }}} ,\;\sigma \left( {K_{{\text{s}}}^{2} } \right) = c_{{K_{{\text{s}}} }} K_{{\text{s}}}^{2} \sqrt {\frac{1}{{w^{2} N_{{{\text{fr}}}} }}} ,\;\sigma \left( {K_{{\text{r}}}^{2} } \right) = c_{{K_{{\text{r}}} }} K_{{\text{r}}}^{2} \sqrt {\frac{1}{{w^{2} N_{{{\text{fr}}}} }}} ,$$

where ${\rm NIO}$ is the number of independent observations, ${N}_{{\rm fr}}$ is the number of frames averaged, ${c}_{{K}_{{s}}}$ and ${c}_{{K}_{{r}}}$ are calibration factors set to 1.90 and 1.47 respectively assuming an exponential intensity distribution for the speckle pattern, and ${w}^{2}$ is the number of pixels imaging the fiber output which has an upper limit equal to the number of pixels on the camera. ${\rm NIO}$ is given by ${\rm NIO}={w}^{2}f\left({\rm s}/{\rm p}\right)$, where $f\left({\rm s}/{\rm p}\right)$ is a factor that accounts for the degree of spatial correlation across pixels as described in more detail here^25^. The SNR is then given by

$${\rm SNR} = {K}_{{\rm f}}^{2}\sqrt{{N}_{{\rm fr}}}/\sigma \left({K}_{{\rm raw}}^{2}\right)$$

### Study participants

One participant within the 20 to 60 year age group with no prior diagnosis or treatment of neurological disorders was recruited for this study. Sex, gender, race, and ethnicity were not considered during recruitment. The participant was recruited through word of mouth on the Boston University campus. The experimental procedure and protocols were approved and carried out in accordance with the regulations of Institutional Review Board of Boston University. All experiments were performed in accordance with relevant guidelines and regulations, including the Declaration of Helsinki. Each participant provided a signed informed consent form prior to the experiment.

## Description of SCOS system hardware used for human CBF measurement

The SCOS system broadly consists of the laser source and the detection setup. Within the laser source, continuous-wave laser light is emitted by an 852 nm laser diode (Thorlabs LD852-SEV600) which is housed in a temperature-controlled laser mount (Thorlabs LDM90) and driven by a combined current and temperature controller (Thorlabs ITC4001). The laser emission from the diode is collimated by an aspheric lens (Thorlabs C110TMD-B) that is attached to the laser mount via an adapter plate (Thorlabs LDMXY). The collimated beam is sent through an optical isolator (Thorlabs IO-5-850-VLP) to protect the diode from back-reflected light. The free-space beam is then blocked periodically by a 10% duty-cycle optical chopper (Thorlabs MC2000B with MC1F2P10 chopper blade) to convert the continuous-wave beam to a pulsed beam. The pulsed beam is then coupled to an optical fiber using a fiber coupler (Thorlabs PAF-X-15-PC-B FiberPort), and the beam is emitted into the subject’s skin by applying the distal end of the optical fiber to the subject’s forehead.

In the detection setup, a custom fiber bundle (~ 3700 strands of 37-µm core diameter multimode fiber, 0.66 NA) collects backscattered light from the subject and transmits it to the camera. The camera end of the fiber bundle is rectangularly shaped (3 × 1.64 mm) to approximately match the aspect ratio of the camera sensor. The fiber bundle image is magnified by a 4-f lens system consisting of an aspheric condenser lens (Thorlabs ACL25416U-B) and a plano-convex lens (Thorlabs LA1027-B). The Basler a2A1920-160umPRO CMOS camera is placed around the focal point of the plano-convex lens. We slightly alter the distance between the fiber bundle and the aspheric condenser lens from the focal length to defocus the fiber bundle image and reduce spatial heterogeneity.

The average illumination power of our system was 38 mW. We calculated the average photon flux per speckle of our system during our human CBF measurement (shown later) as follows. The photon flux per speckle is derived from the mean intensity $\langle I\rangle$ in units of DN from the camera, the camera gain $g$, the camera’s quantum efficiency (QE) at the laser’s wavelength of 852 nm, the exposure time ${T}_{{\rm exp}}$, and the speckle-to-pixel size ratio (s/p) as follows:

$${\rm Photon\, flux\, per\, speckle}=\langle I\rangle /g/{\rm QE}/{T}_{{\rm exp}}\,{\left({\rm s}/{\rm p}\right)}^{2}$$

For our human CBF measurement using the above system $\langle I\rangle =25$ DN, $g=0.58954$ DN/e^-^ as measured from the fit to the photon transfer curve, QE(852 nm) = 0.16 as given in the camera specifications, ${T}_{{\rm exp}}=0.833$ ms, and (s/p)^2^ = 0.7112 as measured using a static phantom. This leads to an instantaneous photon flux per speckle of 2.2619 × 10^5^ s^-1^. As mentioned earlier, we use a 10% duty cycle optical chopper in our system. Therefore, the average photon flux per speckle is a tenth of the instantaneous value above, or 22,619 s^-1^. We use this value in our analytical SCOS noise model later in this work.

### fNIRS and SCOS data acquisition

We used a commercial high-density fNIRS system to locate the activation region on the forehead with the largest task-averaged change in total hemoglobin concentration in response to the mental subtraction task (described in the next section). The commercial fNIRS system (NIRSport2, NIRx) consists of 14 sources and 32 detectors which are placed on the prefrontal cortex region. The source and detector optodes are mounted on a NinjaCap, an in-house 3D printed cap made of a flexible material (NinjaFlex, NinjaTek) that allows for customizable high-density fNIRS measurements with commercial fNIRS systems. The first and second nearest source-detector separation distances are 19 mm and 33 mm respectively. The cap is positioned on the head using anatomical landmarks (nasion, inion, and left/right pre-auricular points), such that the EEG 10–10 midline central electrode site (Cz) is matched between the cap and the head. The signal quality for each source-detector pair was tested through the Aurora fNIRS acquisition software (NIRx) prior to the fNIRS measurement.

For the SCOS measurement, we used the same NinjaCap in order to match the source-detector locations used in the fNIRS measurement. The camera was connected to a computer via a USB 3.0 cable for both power and data acquisition. The camera’s external trigger was used to synchronize both the chopper wheel controller (Thorlabs MC2000B) and the data acquisition card (NI USB-6002). We adjusted the phase of the chopper wheel manually so that the incident laser pulse was contained within the camera’s exposure window.

The data acquisition card also received the trigger signal from a stimulus tracker (Cedrus StimTracker Quad) which indicated the timing of the stimuli to the subject. The stimulus tracker recorded the change in brightness at a corner of the computer display which occurred simultaneously with presentation of stimulus to the subject.

The data acquisition card was connected via micro-USB to the computer and controlled using MATLAB software. The trigger signals from the camera and stimulus tracker were recorded to the computer through the data acquisition card and used later for stimulus-based trial averaging.

### Mental subtraction task protocol

During the mental subtraction task, the participant was seated in front of a computer monitor that provided visual cues. The participant was presented with a cross symbol (+) in the center of the screen during an initial baseline measurement and in between mental subtraction trials. During a mental subtraction trial, the participant was visually presented with a subtraction problem comprising a random three-digit minuend (e.g. 370) and one of three subtrahends (6, 7, or 13). The participant repeatedly subtracted the subtrahend (e.g. 13) from the difference (e.g. 357, 344, 331) until the problem was replaced by the cross symbol on the screen, upon which the participant rested until the next trial. Each problem was displayed for 20 s and the interval between problems was randomly varied between 20 and 25 s. Each “run” of the mental subtraction task consisted of five trials. Each participant performed a total of 3 runs (15 trials in total) which lasted 12 min. No feedback regarding the measurement was given to the participant during and after the measurement.

### Statistical information

A two-sample, two-tailed t-test was used to determine whether the changes in optical density (OD) and blood flow index (BFi) measured during 15 trials of the mental subtraction task were statistically significant. For both ΔOD and ΔBFi, 15 values—one from each trial—were used at the beginning of the trial (time = 0 s) and 15 values were used at the maximum trial-averaged value (time ≈ 13.5 s for ΔOD and time ≈ 10.5 s for ΔBFi). ΔOD: *p* = 2.04 × 10^–7^, t = 6.83, df = 28. ΔBFi: *p* = 1.06 × 10^–5^, t = 5.35, df = 28.

## Results

**Figure 2:**
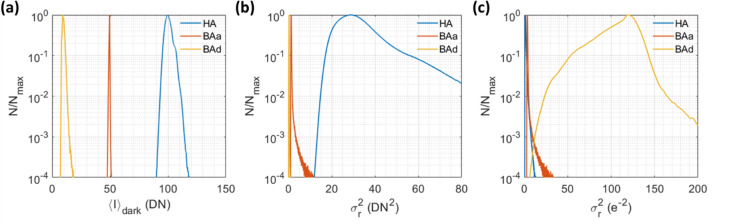
Dark offset and read noise variance distributions for the three cameras. (**a**) Normalized mean dark offset ${\langle I\rangle }_{{\rm dark}}$ distributions across pixels for the three cameras in units of DN. Here $N$ is the number of pixels at a particular dark offset, and it is normalized by its maximum value ${N}_{{\rm max}}$. The mean dark offsets of the cameras are HA = 100.5 DN, BAa = 49.4 DN, and BAd = 9.7 DN. (**b**) Normalized read noise variance ${\sigma }_{{r}}^{2}$ distributions across pixels for the three cameras in units of DN^2^. The RMS read noise values of the cameras are HA = 5.99 DN, BAa = 1.16 DN, and BAd = 0.48 DN. (**c**) Normalized read noise variance ${\sigma }_{{r}}^{2}$ distributions for the three cameras in units of e^−2^. The RMS read noise values of the cameras are HA = 1.28 e^−^, BAa = 1.97 e^−^, and BAd = 10.33 e^−^.

In SCOS, the speckle contrast $K=\sigma \left(I\right)/\langle I\rangle$ is calculated, where $I$ is the speckle intensity measured by the camera in units of the camera’s digital output or digital numbers (DN), $\sigma \left(\dots \right)$ denotes the standard deviation, and $\langle \dots \rangle$ denotes the average over pixels. A noise correction procedure has been developed^26^ to remove the biases in $\sigma \left(I\right)$ and $\langle I\rangle$, which requires accurate measurements of camera gain $g$, pixel-wise mean dark offset ${\langle I\rangle }_{{\rm dark}}$, and pixel-wise read noise ${\sigma }_{{\rm r}}$. Camera nonidealities can introduce errors in the estimated values of $g$, ${\langle I\rangle }_{{\rm dark}}$, and ${\sigma }_{{\rm r}}$ Fig. 2. Thus, it is important to characterize and assess the impact of the nonidealities when choosing a camera for SCOS.

Our camera selection and system optimization procedure for SCOS is summarized as follows:

Camera selection:

- Step 1. Measure the camera’s photon transfer curve, defined as the variance of the intensity ${\sigma }^{2}\left(I\right)$ versus the average intensity $\langle I\rangle$ (See Methods section for details):
  - Estimate the camera’s gain $g$ from the slope of the linear fit to the photon transfer curve
  - Evaluate the camera nonlinearity in the photon transfer curve and calculate its impact on SCOS measurements
- Step 2. Measure the camera’s mean dark offset ${\langle I\rangle }_{{\rm dark}}$ and read noise ${\sigma }_{{\rm r}}$ distributions across pixels (See Methods section for details):
  - Determine whether there is quantization distortion that could prevent accurate measurement of the camera’s ${\langle I\rangle }_{{\rm dark}}$ and ${\sigma }_{{\rm r}}$ values in units of DN
  - Using the measured camera gain $g$ from Step 1, convert from units of DN to electrons (e^-^) in order to compare the ${\sigma }_{{\rm r}}$ distributions between different cameras
- Step 3. Downselect cameras based on their properties including photon transfer curve linearity, read noise level, camera gain value, quantization distortion, and cost.
  Camera and system parameter optimization:
- Step 4. Using the camera parameters obtained from characterization, find the optimal camera exposure time ${T}_{{\rm exp}}$ and speckle-to-pixel size ratio s/p for maximal SNR using the SCOS noise model^25^:
  - If the laser source can be pulsed, optimize the laser’s peak power and duty cycle in conjunction with the ${T}_{{\rm exp}}$ and s/p for maximal SCOS SNR
  - Repeat this process if desired for multiple cameras to compare their achievable SNRs in SCOS

## Camera selection

The basic camera characterization process for SCOS consists of measuring the camera gain $g$, pixel-wise dark offset ${\langle I\rangle }_{{\rm dark}}$, and pixel-wise read noise ${\sigma }_{{\rm r}}$. The purpose of this process, apart from acquiring the parameters needed for SCOS noise correction, is to detect any presence of camera nonidealities that are not corrected for by our current SCOS noise correction procedure^26^ due to their complicated dependence on intensity or temporal fluctuation. These nonidealities include the intensity dependence in $g$ and the distortion in the measured ${\langle I\rangle }_{{\rm dark}}$ and ${\sigma }_{{\rm r}}$ due to insufficiently high $g$, and they impact SCOS measurement accuracy We illustrate this process for the three commercially available CMOS cameras mentioned in the Introduction (HA, BAa, BAd).

### Step 1: Measure the camera’s photon transfer curve

The schematic of the camera characterization setup is shown in Fig. 1a. In Fig. 1b–d, we show the photon transfer curve for each camera, measured using uniform incoherent illumination with mean intensities $\langle I\rangle$ spanning the full bit depth of each camera. The slope of the linear fit to the photon transfer curve is the camera gain value $g$ which is needed to correct for the bias in $K$ introduced by shot noise in SCOS measurements. A camera’s gain may be adjustable and is generally influenced by other camera settings such as bit depth. Therefore, the gain value reported in a camera datasheet may not be applicable to the user’s specific camera settings and we recommend that the user obtain $g$ from their own photon transfer curve measurement.

We see in Fig. 1b that the photon transfer curve for the more costly scientific CMOS camera, HA, exhibits sharp deviations from the linear fit while the lower-cost CMOS cameras, BAa (Fig. 1c) and BAd (Fig. 1d), are more linear. The linearity is quantified by the linearity error, calculated as the mean magnitude of the relative deviation from the linear fit $\langle \left|\left(y-\widehat{y}\right)/\widehat{y}\right|\rangle$, where $y$ is the experimentally obtained data and $\widehat{y}$ is the fitting result. All three cameras deviate from linear behavior at high DN values near saturation, and thus that region should be avoided when conducting SCOS measurements. For human brain measurements, the photon flux is usually low enough to avoid the saturation region of the cameras.

Systematic nonlinearity in the camera’s photon transfer curve can induce errors in the measured speckle contrast $K$. This occurs because such nonlinearities produce deviations from the anticipated bias in $K$ induced by shot noise. In Fig. 1e–g, we show the error in ${K}^{2}$ induced by this systematic nonlinearity for all three cameras as a function of $\langle I\rangle$. We estimate the magnitude of the systematic error in ${K}^{2}$ due to the systematic nonlinearities as $\left|\Delta {K}^{2}\right|=\left|\left[{\sigma }^{2}\left(I\right)-{\sigma }_{{\rm fit}}^{2}\left(I\right)\right]/{\langle I\rangle }^{2}\right|$, where ${\sigma }_{{\rm fit}}^{2}\left(I\right)$ is the variance of the intensity predicted from the linear fit to the photon transfer curve. Because $\Delta {K}^{2}$ is inversely related to ${\langle I\rangle }^{2}$, deviations in ${\sigma }^{2}\left(I\right)$ from the linear fit have larger impacts on $\left|\Delta {K}^{2}\right|$ at lower $\langle I\rangle$ values which are more relevant to human brain measurements. Thus, we scale the relative error in ${\sigma }^{2}\left(I\right)$ by $1/\langle I\rangle$ to increase the weighting at lower $\langle I\rangle$ values and minimize the squared sum of the scaled relative errors when deriving the linear fit to the photon transfer curve.

Given the intensity-dependence of the camera gain value $g$, which manifests as nonlinearity in the photon transfer curve (Fig. 1b–d), the fitting of the photon transfer curve to estimate $g$ should be performed within the user’s operating intensity range to maximize the accuracy of the SCOS noise correction (shaded region in Fig. 1b–d). In this work, we chose a lower operating limit corresponding to the $\langle I\rangle$ at which shot noise is equal to twice the camera’s read noise, to favor operating in the shot-noise-limited regime. We chose an upper operating limit corresponding to 70% of the camera’s saturation intensity, ${\langle I\rangle }_{{\rm sat}}$, defined as the $\langle I\rangle$ at which the intensity histogram from an image with temporal noise included starts to become clipped at the camera’s maximum intensity value. ${\langle I\rangle }_{{\rm sat}}$ corresponds to the beginning of the plateau in the first-order response, defined as the mean intensity $\langle I\rangle$ versus the illumination power $\Phi$.. We chose this upper operating limit to avoid the roll-off of the photon transfer curve near saturation.

We see that the nonlinearity-induced $\left|\Delta {K}^{2}\right|$ within the chosen operating intensity range can be as high as 2 × 10^–3^ for the HA and 7 × 10^–4^ for the BAa, which are 10% and 3.5% respectively of the average ${K}_{{\rm f}}^{2}$ value (~ 2 × 10^–2^) from our human CBF measurement (shown later). In addition, the abrupt nonlinearities in the HA’s photon transfer curve cause sharp peaks in $\left|\Delta {K}^{2}\right|$ between certain $\langle I\rangle$ values. The BAd shows a comparatively low maximum $\left|\Delta {K}^{2}\right|$ of 1.5 × 10^–5^ within the chosen operating intensity range, but it should be noted that a much longer integration time would be required to reach its lower operating limit in a human CBF measurement due to its high read noise (shown later).

Two options are available for reducing the impact of the nonlinearity-induced $\left|\Delta {K}^{2}\right|$ on SCOS measurement accuracy. The first is to ensure that $\left|\Delta {K}^{2}\right|$ is negligible compared to the measured “fundamental” blood-flow-induced contrast squared, ${K}_{{\rm f}}^{2}$, within the $\langle I\rangle$ range used in the measurement. Since $\Delta {K}^{2}$ is roughly inversely related to ${T}_{{\rm exp}}^{2}$ through ${\langle I\rangle }^{2}$ while ${K}_{{\rm f}}^{2}$ is inversely related to ${T}_{{\rm exp}}$, $\left|\Delta {K}^{2}\right|$ can be decreased relative to ${K}_{{\rm f}}^{2}$ by increasing ${T}_{{\rm exp}}$. Another option is to correct for the nonlinearity-induced $\left|\Delta {K}^{2}\right|$, which in principle can be done if $g$ is characterized precisely over the operating intensity range. Correction of the nonlinearity-induced $\left|\Delta {K}^{2}\right|$ however is complicated by the fact that the speckle pattern and the illumination pattern contain a distribution of $\langle I\rangle$ values in contrast to the uniform incoherent (LED) illumination used to characterize a camera.

### Step 2: Measure the camera’s mean dark offset ${\langle {\varvec{I}}\rangle }_{{\varvec{d}}{\varvec{a}}{\varvec{r}}{\varvec{k}}}$ and read noise ${{\varvec{\sigma}}}_{{\varvec{r}}}$ distributions across pixels

The per-pixel mean dark offset $${\langle I\rangle }_{{\rm dark}}\left(x,y\right)$$ and read noise variance $${\sigma }_{{\rm r}}^{2}\left(x,y\right)$$ for a camera are obtained by calculating the temporal mean and variance, respectively, for every pixel across a set of dark images. In the SCOS noise correction procedure, the $${\langle I\rangle }_{{\rm dark}}\left(x,y\right)$$ image is subtracted from every raw image to correct for the camera’s dark offset nonuniformity, while the $${\sigma }_{{\rm r}}^{2}\left(x,y\right)$$ image is used to calculate the read-noise-induced contrast term, $${K}_{{\rm r}}^{2}={\sigma }_{{\rm r}}^{2}/{\langle I\rangle }^{2}$$. Shown in Fig. 2a, b are the $${\langle I\rangle }_{{\rm dark}}$$ and $${\sigma }_{{\rm r}}^{2}$$ distributions across pixels, respectively, for the three cameras in units of DN. To compare read noise distributions between cameras, the read noise values in units of DN need to be converted to units of electrons (e^−^) by dividing the DN values by the previously characterized camera gain $$g$$ (DN/e^-^). Figure 2c shows the $${\sigma }_{{\rm r}}^{2}$$ distributions for the three cameras in units of e^-2^. We see that the HA and BAa have similar root-mean-square (RMS) read noise values (HA: 1.28 e^-^, BAa: 1.97 e^-^), despite the HA costing considerably more (~ $26,000 vs. ~ $500 at the time of publication). The BAd has much higher (> 5x) RMS read noise (10.33 e^-^) than the other two cameras. Besides lowering SNR, the higher read noise would require us to use a much longer (> 25x) camera integration time with the BAd to achieve the same shot-noise-limited SCOS SNR during human CBF measurements at long source-detector separation, limiting the acquisition rate as compared to what is achievable with other cameras.

Note that while an RMS read noise value is typically reported in a camera’s datasheet, a camera’s read noise is generally influenced by other camera settings such as analog gain and bit depth. In addition, since a CMOS camera uses an independent amplifier for each pixel, there is read noise variation across pixels that needs to be quantified for our SCOS noise correction procedure. For these reasons, the user should perform a per-pixel read noise (and dark offset) measurement with the camera settings that they would like to use for SCOS. Obtaining the read noise for all pixels also enables the identification of outlier pixels with highly elevated read noise. The user can choose to exclude these pixels from analysis in the SCOS noise correction procedure.

Although the BAd has the largest mean read noise variance ${\sigma }_{{\rm r}}^{2}$ in units of e^-2^, the ${\sigma }_{{\rm r}}^{2}$ distribution for BAd in Fig. 2b is almost entirely below 1 DN^2^ and has a mean of 0.23 DN^2^, with many pixels showing near-zero ${\sigma }_{{\rm r}}^{2}$. (The BAa and HA cameras have mean ${\sigma }_{{\rm r}}^{2}$ values of 1.3 DN^2^ and 35.9 DN^2^ respectively.) Because of the BAd’s low camera gain value (Fig. 1d), its measured per-pixel dark offset ${\langle I\rangle }_{{\rm dark}}\left(x,y\right)$ and read noise ${\sigma }_{{\rm r}}\left(x,y\right)$ values are distorted by quantization from the true ${\langle I\rangle }_{{\rm dark}}\left(x,y\right)$ and ${\sigma }_{{\rm r}}\left(x,y\right)$ values, respectively.

Quantization occurs when a pixel’s analog voltage reading is converted to a digital integer value, and it is another source of noise that affects SCOS measurements. For instance, assuming that a rounding operation is implemented in the camera, an intensity reading corresponding to an unquantized value of 4.1 will be rounded to 4, generating an error of 0.1. In principle, observing multiple realizations of the quantized signal will allow for recovering the unquantized mean intensity and the variance, provided that there is sufficiently large variance in the observations. Quantization distortion refers to the error in the recovered mean and variance, which becomes non-negligible when the signal variance in units of camera digital numbers (DN^2^) becomes small relative to 1 DN. To explore the effect of quantization distortion, we numerically drew numbers from Gaussian distributions with certain mean and variance values, and then estimated the mean and variance from the quantized numbers (See Methods).

**Figure 3:**
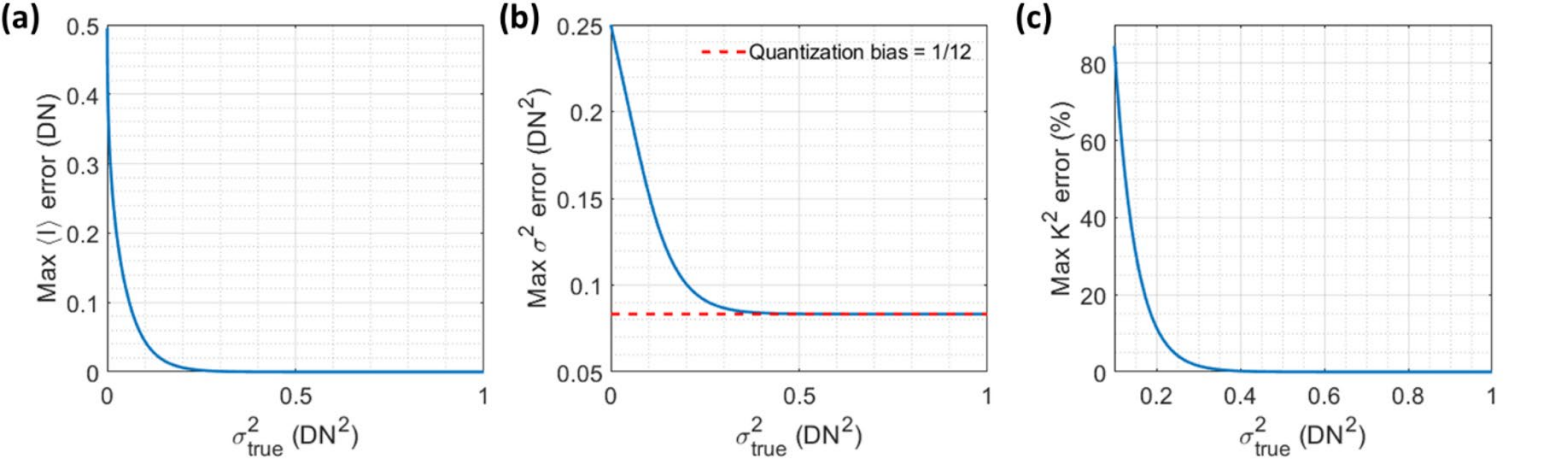
Impact of quantization distortion on SCOS measurement accuracy. (**a**) Maximum quantization-distortion-induced error in measured mean intensity $\langle I\rangle$ as a function of the true variance ${\sigma }_{{\rm true}}^{2}$. (**b**) Maximum quantization-distortion-induced error in measured variance ${\sigma }^{2}$ as a function of the true variance ${\sigma }_{{\rm true}}^{2}$. The error in ${\sigma }^{2}$ asymptotically approaches 1/12 (red dashed line) with increasing ${\sigma }_{{\rm true}}^{2}$. (**c**) Corresponding error in ${K}^{2}$ given the errors in $\langle I\rangle$ (**a**) and ${\sigma }^{2}$ (**b**) as a function of ${\sigma }_{{\rm true}}^{2}$, after subtracting an assumed quantization-induced bias of 1/12 from the signal’s variance.

Figure 3a, b show the maximum quantization-distortion-induced error in the estimated mean intensity $\langle I\rangle$ and variance ${\sigma }^{2}$, respectively, as a function of the true variance ${\sigma }_{{\rm true}}^{2}$ of the simulated Gaussian distributions. They show that below a variance of 0.2 DN^2^, which applies to a significant fraction of the pixels in the BAd’s dark images, the maximum quantization-induced errors in both the estimated $\langle I\rangle$ and ${\sigma }^{2}$ are no longer negligible. This causes the subtraction of the ${\langle I\rangle }_{{\rm dark}}$ pattern and the read-noise-induced contrast term ${K}_{{\rm r}}^{2}$ to be inaccurate in SCOS measurements with the BAd, namely due to its low camera gain value. The impact of the quantization distortion on SCOS measurements is expressed in Fig. 3c as the maximum percent error in ${K}^{2}$ as a function of ${\sigma }_{{\rm true}}^{2}$. We see that above ${\sigma }_{{\rm true}}^{2}$ = 0.4 DN^2^, the quantization-induced error in ${K}^{2}$ is negligible after subtracting the quantization-induced bias of 1/12^31,32^ from the measured ${\sigma }^{2}$. Subtraction of the quantization variance bias is done in the SCOS noise correction procedure as described in the Methods. We recommend using a camera with a high enough gain value such that the camera’s ${\sigma }_{{\rm r}}^{2}$ distribution is entirely above 0.4 DN^2^ where quantization distortion becomes negligible. We note that some cameras like the BAd only allow for increasing the post-quantization or “digital” gain which multiplies the DN values by a factor ${d}_{{\rm g}}$. This does not mitigate quantization distortion because it simply multiplies the measured mean and variance by ${d}_{{\rm g}}$ and ${d}_{{\rm g}}^{2}$ while effectively increasing the quantization interval size in DN by the same factor ${d}_{{\rm g}}$.

### Step 3: Downselect cameras

With the results from this camera characterization process, we decided that the BAd (~ $200 at the time of purchase) is not a preferred camera for SCOS among the three because it suffers from quantization-distortion-induced $${K}^{2}$$ error and has significantly higher (> 5x) RMS read noise than the other two cameras, which would limit acquisition rate and SNR in SCOS. The HA is considerably more expensive (~ $26,000 at the time of purchase) than the other two cameras and has the highest nonlinearity within the chosen operating limits for the cameras, which results in the HA suffering from relatively large peaks in nonlinearity-induced systematic $${K}^{2}$$ error. While during a measurement with the HA one could in principle avoid the intensity ranges where the peaks in systematic $${K}^{2}$$ error are located, in practice that may be difficult due to intra- and inter-subject variability and the existence of an intensity distribution in the illumination and speckle patterns. Hence, the HA is not a preferred option either for SCOS. Since the BAa (~ $500 at the time of purchase) has negligible quantization distortion, comparable read noise level to the HA, and lower peaks in nonlinearity-induced $${K}^{2}$$ error than the HA within the chosen operating range, we considered it the best camera choice among the original three for SCOS.

## Camera and system parameter optimization

Once cameras under consideration are characterized and those with negligible nonlinearity and quantization distortion are identified, the next step is to optimize the SCOS system operating parameters for maximal SNR. The parameters having the largest influence on SCOS measurement SNR are the exposure time ${T}_{{\rm exp}}$ and speckle-to-pixel size ratio s/p. Since we have previously identified the BAa as our preferred camera in the camera selection process, we will use it to illustrate the concepts in the system parameter optimization process.

**Figure 4:**
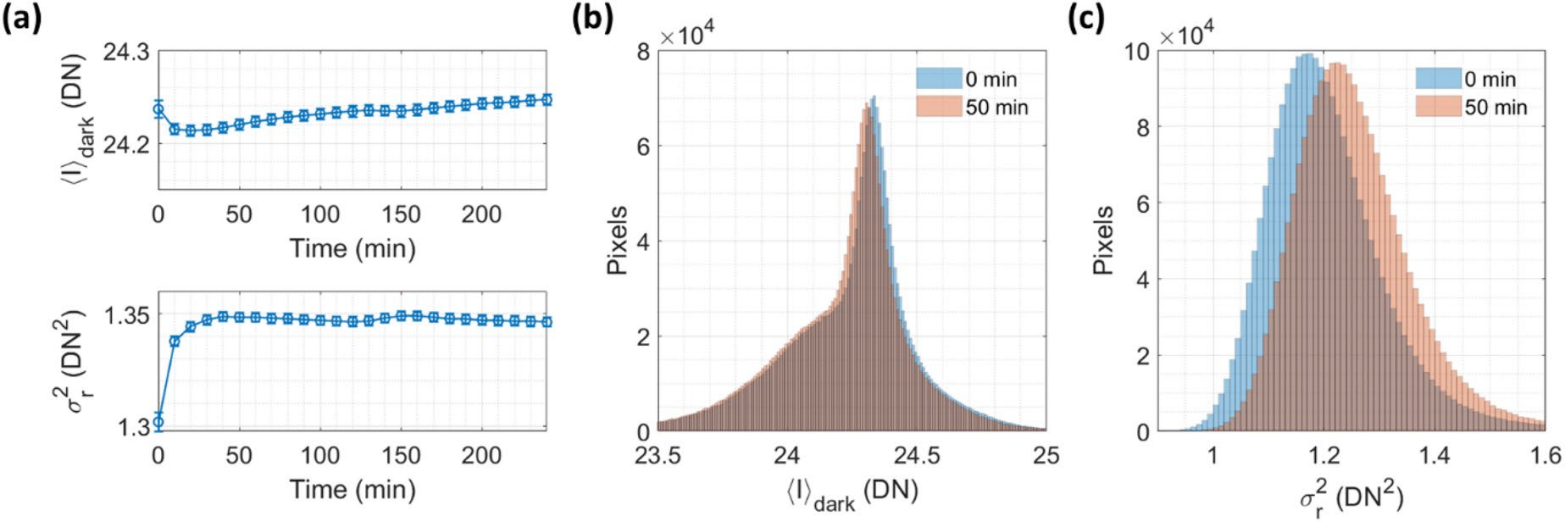
Dark offset and read noise variability of the BAa camera. (**a**) Time course of pixel-averaged mean dark offset ${\langle I\rangle }_{{\rm dark}}$ and read noise variance ${\sigma }_{{r}}^{2}$. Dark measurements were performed every 10 min, and prior to the first measurement the camera was powered on but not acquiring images. Error bars represent the temporal standard deviation of the mean of each image (top) and the variance of each difference image (bottom). (**b**) Change in the ${\langle I\rangle }_{{\rm dark}}$ distribution across pixels between the two time points indicated. (**c**) Change in the ${\sigma }_{{r}}^{2}$ distribution across pixels between the same two time points.

In practice, we adjust ${T}_{{\rm exp}}$ and s/p to minimize read noise contributions to the measured speckle contrast. More specifically, we desire to make measurements in the shot-noise-limited regime in which the read-noise-induced contrast squared ${K}_{{\rm r}}^{2}={\sigma }_{{\rm r}}^{2}/{\langle I\rangle }^{2}$ is much smaller than the shot-noise-induced contrast squared ${K}_{{\rm s}}^{2}=g/\langle I\rangle$. A challenge of operating in a regime where the contribution of read noise is non-negligible is that the read noise can vary in time, as we illustrate in Fig. 4. Recall that in Fig. 2 we showed examples of per-pixel mean dark offset ${\langle I\rangle }_{{\rm dark}}\left(x,y\right)$ and read noise variance ${\sigma }_{{\rm r}}^{2}\left(x,y\right)$ measured from ~ 10 s of data acquisition. To explore the stability of the ${\langle I\rangle }_{{\rm dark}}$ and ${\sigma }_{{\rm r}}^{2}$ distributions over a longer timescale (> 10 min), we measured the pixel-averaged ${\langle I\rangle }_{{\rm dark}}$ and ${\sigma }_{{\rm r}}^{2}$ values from the BAa camera every 10 min for 4 h as shown in Fig. 4a. The camera was powered on but not acquiring images for 3 h prior to the first measurement, while in between measurements the camera continued to acquire images but did not save them. We see that ${\langle I\rangle }_{{\rm dark}}$ and ${\sigma }_{{\rm r}}^{2}$ varied over time, possibly due to environmental factors such as fluctuations in the camera’s temperature (we observed this behavior for all three cameras). This effect was more pronounced immediately after the camera started acquiring images from the first measurement, presumably because the process of acquiring images caused the camera’s temperature to increase until reaching a new steady state. This effect can impact the accuracy of noise correction in the low-photon-flux regime where the contribution of read noise is non-negligible. Figure 4b, c show the variations in the per-pixel ${\langle I\rangle }_{{\rm dark}}$ and ${\sigma }_{{\rm r}}^{2}$ respectively. To reduce the impact of inaccuracy in the calculated read-noise-induced speckle contrast ${K}_{{\rm r}}$ due to the camera’s read noise variability, we recommend avoiding operating regimes where ${K}_{{\rm r}}^{2}$ is dominant. We also recommend the practice of updating the recorded per-pixel ${\langle I\rangle }_{{\rm dark}}\left(x,y\right)$ and ${\sigma }_{{\rm r}}^{2}\left(x,y\right)$ before each SCOS measurement for more accurate noise correction. The results in Fig. 4a suggest that having the camera “warm up” by acquiring images for some time prior to conducting a SCOS measurement may reduce the read noise variation during the measurement.

### Step 4: Using the camera parameters obtained from characterization, find the optimal camera exposure time and s/p for maximal SNR

**Figure 5:**
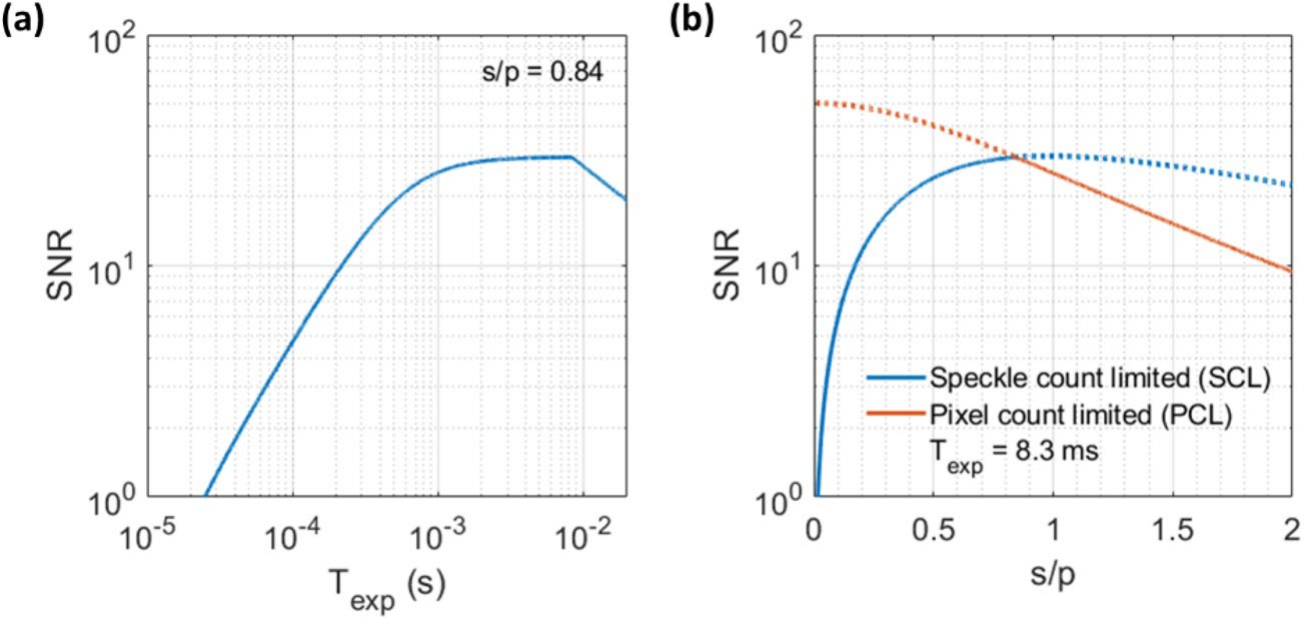
SCOS SNR dependence on exposure time and s/p ratio for the BAa camera. (**a**) SNR versus ${T}_{{\rm exp}}$ for s/p = 0.84. (**b**) SNR versus s/p at ${T}_{{\rm exp}}$ = 8.3 ms for speckle-count-limited and pixel-count-limited cases. When the SCOS system is speckle-count-limited, the total number of speckles/fiber modes is fixed at M = $3.3\times {10}^{6}$, estimated from the area of the fiber output image and the s/p ratio obtained experimentally. In the speckle-count-limited case we assume that the camera has enough pixels to image all the speckles. When the system is pixel-count-limited, the total number of pixels is fixed at 1936 × 1216 pixels for the BAa camera, and we assume all pixels are filled with speckles. The SNR vs. s/p of our fiber-based SCOS system follows the solid red and blue lines. BAa camera parameter values of 1.97 e^−^ RMS read noise, 16% quantum efficiency (QE) at 852 nm wavelength, 120 Hz maximum frame rate (${f}_{{\rm max}}$) at 10-bit depth, and 1936 × 1216 pixels were used. An average photon flux per speckle of 22,619 s^-1^ (as derived in the Methods) and a measurement rate of 10 Hz were used.

Having established that the user should try to operate in the shot-noise-limited regime, we now discuss how to accomplish that and maximize SCOS SNR by choosing ${T}_{{\rm exp}}$ and s/p. We define SNR in SCOS as SNR = ${K}_{{\rm f}}^{2}/\sigma \left({K}_{{\rm raw}}^{2}\right)$, where $\sigma \left({K}_{{\rm raw}}^{2}\right)$ is the root-sum-square of the noise from individual ${K}^{2}$ terms. We use our recently developed and validated SCOS noise model^25^ as described in the Methods to predict the SNR as functions of ${T}_{{\rm exp}}$ and s/p in Fig. 5, assuming a photon flux per speckle of 22,619 s^-1^ as obtained on average during our human CBF measurement (shown later) and a measurement interval time of 100 ms over which the measured ${K}_{{\rm f}}^{2}$ values from individual camera frames are averaged. In addition to the RMS read noise characterized previously, the noise model uses the following camera parameters: quantum efficiency (QE) at the operating wavelength, maximum frame rate (${f}_{{\rm max}}$), and total number of pixels. Those camera parameters are readily obtained from the camera’s datasheet. For reference, the BAa camera has a QE of 16% at 852 nm wavelength, ${f}_{{\rm max}}$ of 120 Hz at 10-bit depth, and 1936 × 1216 pixels.

We see in Fig. 5a that, for a given s/p determined by the optical setup and the camera’s pixel size, SNR = ${K}_{f}^{2}/\sigma \left({K}_{{\rm raw}}^{2}\right)$ plateaus at a particular ${T}_{{\rm exp}}$ which indicates the beginning of the shot-noise-limited regime where shot-noise-induced contrast ${K}_{{\rm s}}$ dominates and the impact of camera read noise becomes negligible. The plateauing in SNR occurs because both ${K}_{{\rm f}}^{2}$ and noise in the measured ${K}_{{\rm s}}^{2}$, i.e. $\sigma \left({K}_{{\rm s}}^{2}\right)$ which is the dominant term in $\sigma \left({K}_{{\rm raw}}^{2}\right)$ under the shot-noise-limited regime, are inversely proportional to ${T}_{{\rm exp}}$. The reduction in SNR for ${T}_{{\rm exp}}$ > 8.3 ms is due to a decrease in the camera’s frame rate and consequent reduction in frame averaging within the 100 ms measurement interval time. This happens because ${T}_{{\rm exp}}$ becomes larger than 1/${f}_{{\rm max}}$. For the SNR’s dependence on s/p value (Fig. 5b), we show the cases where SNR is limited by the number of speckles assuming an unlimited number of pixels (speckle-count-limited), and where it is limited by the number of pixels assuming an unlimited number of speckles (pixel-count-limited). We see that for the speckle-count-limited case, there exists an optimal s/p ratio that maximizes SNR for a given ${T}_{{\rm exp}}$ and photon flux per speckle of 22,619 s^-1^. Experimentally, this case corresponds to a fiber-based SCOS system where the number of fiber modes is fixed. If there are not enough pixels on the camera to capture all the speckles/modes, we need to consider the pixel-count-limited case. We see that in such a case, a small s/p is preferred to maximize SNR, as more speckles are imaged on the same camera. For a fiber-based SCOS system utilizing a camera with a finite number of pixels and a rectangular fiber bundle whose aspect ratio matches that of the camera sensor, the SNR dependence on s/p follows the lower of the two SNR curves (solid red and blue lines) at any given s/p value in Fig. 5b. When the fiber output image is totally contained within the camera’s pixel array, this corresponds to the speckle-count-limited case. When the fiber output image is enlarged beyond the bounds of the pixel array, this corresponds to the pixel-count-limited case.

The intersection point of the two curves, where the fiber’s output is matched to the size of the camera’s pixel array, optimally balances the s/p and the number of speckles sampled and gives the maximum SNR in our system with the BAa camera. For cameras with higher read noise, the maximum SNR might be achieved when s/p is reduced such that the fiber’s output does not fill the entire pixel array (See supplementary Fig. S2). This is because reducing the s/p increases the photon flux per pixel and helps the system achieve shot-noise-limited performance. In practice, the user should determine the optimal s/p for their experimental setup by obtaining the required input parameters to the SCOS noise model^25^ (photon flux per speckle, camera parameters, etc.) and running the model as illustrated in Fig. 5.

**Figure 6:**
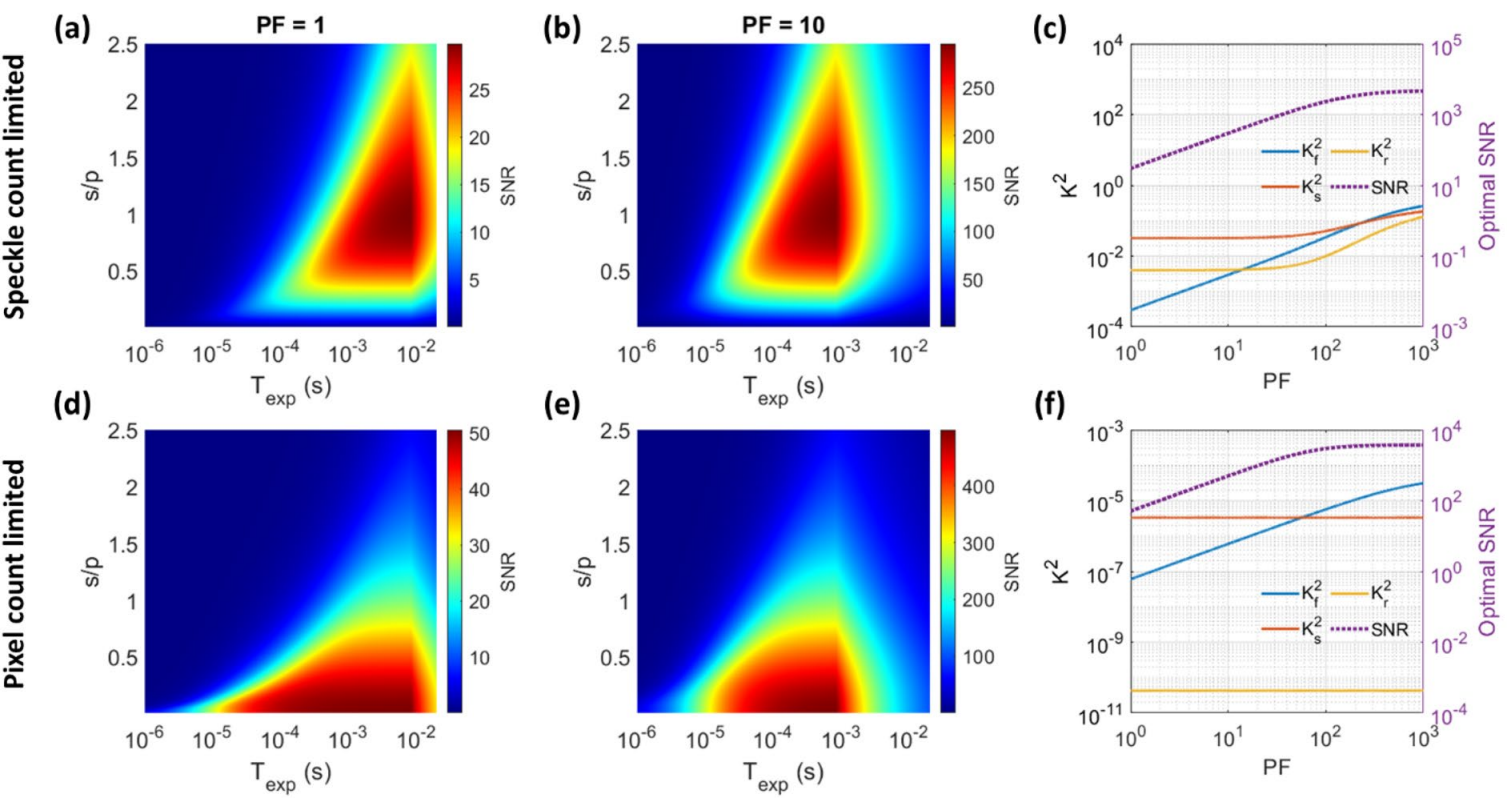
Impact of varying the laser pulsing factor on SCOS SNR with the BAa camera. (**a**–**b**) SNR as a function of s/p and ${T}_{{\rm exp}}$ at (**a**) PF = 1 and (**b**) PF = 10 for the speckle-count-limited case. The total number of speckles/fiber modes is fixed at M = $3.3\times {10}^{6}$ as done in Fig. 5, and we assume the camera has enough pixels to image all the speckles. (**d**–**e**) SNR as a function of s/p and ${T}_{{\rm exp}}$ at (d) PF = 1 and (e) PF = 10 for the pixel-count-limited case. The total number of pixels is fixed at 1936 × 1216 pixels, and we assume all pixels are filled with speckles. The optimal SNR and corresponding ${K}^{2}$ terms are plotted as a function of laser PF for both the (**c**) speckle-count-limited case and (**f**) pixel-count-limited case. In both cases, the SNR plateaus as the blood-flow-induced speckle contrast ${K}_{{\rm f}}^{2}$ dominates and the fundamental noise $\sigma \left({K}_{{\rm f}}^{2}\right)$ becomes the dominant noise source. BAa camera parameter values of 1.97 e^−^ RMS read noise, 16% quantum efficiency (QE) at 852 nm wavelength, 120 Hz maximum frame rate (${f}_{{\rm max}}$) at 10 bit depth, and 1936 × 1216 pixels were used. An average photon flux per speckle of 22,619 s^-1^ (as derived in the Methods) and a measurement rate of 10 Hz were used.

We have discussed that operating in the shot-noise-limited regime is recommended for more accurate and higher-SNR SCOS measurements. In Fig. 5, we illustrated how to achieve shot-noise-limited performance by adjusting ${T}_{{\rm exp}}$ and s/p. Another way to achieve shot-noise-limited performance and further improve SNR is to increase the peak optical power within the camera exposure time while keeping the average power within the safety limit for human measurements^26^. To describe this, we introduce another parameter which is the laser pulsing factor (PF), defined as the inverse of the duty cycle of a pulsed laser source. The laser’s peak power can be increased by the PF while maintaining the same average power, with the peak and average power within the safety limit. We show in Fig. 6 examples of the SNR calculated as functions of s/p and ${T}_{{\rm exp}}$ for PF = 1 and PF = 10 for both the speckle-count-limited case (Fig. 6a, b) and the pixel-count-limited case (Fig. 6d, e). We see that increasing the PF generally increases the SNR by the same factor (except in the fundamental-noise-limited regime), namely through the reduction in ${T}_{{\rm exp}}$ by the PF which increases ${K}_{{\rm f}}^{2}\propto 1/{T}_{{\rm exp}}$ by the same factor while maintaining ${K}_{{\rm s}}^{2}\propto 1/\left({c}_{{\rm p}}{T}_{{\rm exp}}\right)$ and ${K}_{{\rm r}}^{2}\propto 1/{\left({c}_{{\rm p}}{T}_{{\rm exp}}\right)}^{2}$ at the same value, where ${c}_{{\rm p}}$ is the photon flux per pixel. Note that at a higher PF, since the duty cycle is reduced, the acquisition rate could be impacted when ${{\rm PF}\cdot T}_{{\rm exp}}$ becomes larger than 1/${f}_{{\rm max}}$.

Figure 6c, f show that the optimal SNR, i.e. the maximum SNR achieved for the BAa camera at a given PF across all s/p and ${T}_{{\rm exp}}$ values tested, increases linearly with PF until it plateaus in the fundamental-noise-limited regime, where ${K}_{{\rm f}}^{2}$ dominates all other speckle contrast components. In practice, PF is limited by the availability of high-peak-power pulsed laser sources and laser safety limits. In both the speckle-count-limited and pixel-count-limited cases, the optimal way of increasing the PF while maintaining the same average power is by decreasing ${T}_{{\rm exp}}$ while maintaining the same frame rate. We see in the speckle-count-limited case in Fig. 6c that, near the fundamental-noise-limited regime, ${K}_{{\rm s}}^{2}$ and ${K}_{{\rm r}}^{2}$ increase because the optimal s/p has changed. In contrast, for the pixel-count-limited case, SNR is maximized by keeping the s/p at the minimum achievable value (s/p = 0.01 in this simulation) to maximize the number of speckles sampled.

Since blood-flow-induced speckle contrast ${K}_{{\rm f}}$ decreases linearly with decreasing s/p at s/p $\ll$ 1, one may wonder if the spatial contrast would become too small to resolve from the camera’s quantized output at very low s/p. However, since ${\langle I\rangle }^{2}\propto 1/{\left({\rm s}/{\rm p}\right)}^{4}$ and ${K}_{{\rm f}}^{2}\propto {\left({\rm s}/{\rm p}\right)}^{2}$ for s/p $\ll 1$, the fundamental signal’s variance ${\sigma }_{{\rm f}}^{2}$ increases with decreasing s/p as ${\sigma }_{{\rm f}}^{2}\left(I\right)\propto 1/{\left({\rm s}/{\rm p}\right)}^{2}$ and quantization distortion does not become a problem. The only limit to decreasing s/p would be the camera’s saturation limit and the total number of speckles available.

**Figure 7:**
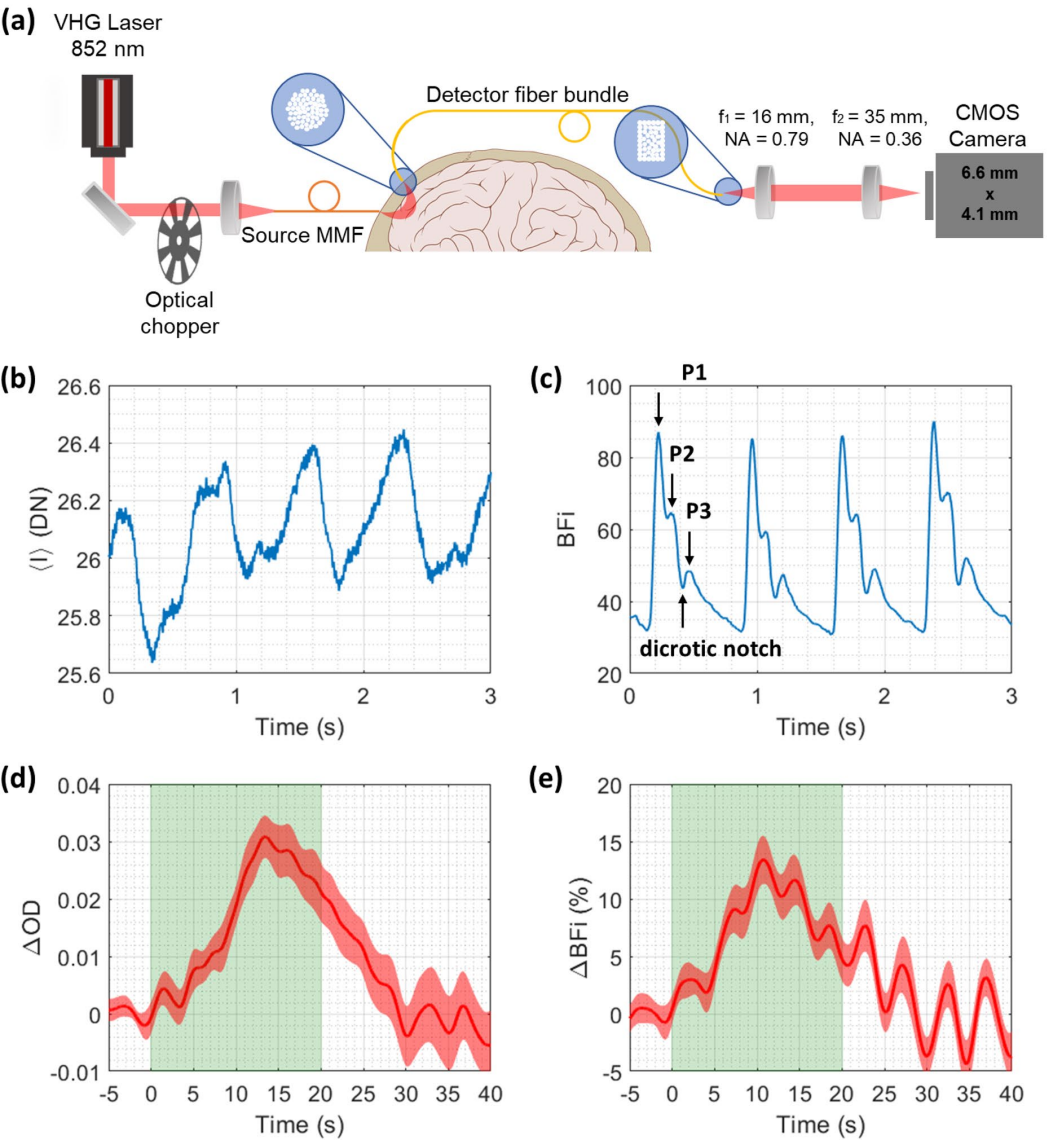
Cardiac and mental subtraction measurements using the BAa camera with optimal operating parameters. (**a**) Diagram of the SCOS measurement setup. Brain and skull image is obtained from Wikimedia Commons. All the other elements of the image are generated by the authors using Microsoft PowerPoint. (**b**–**c**) Recovery of the pulsatile waveform in (**b**) mean intensity $\langle I\rangle$ and (c) BFi = $1/{K}_{{\rm f}}^{2}$ using SCOS. The three peaks (P1, P2, P3) and dicrotic notch are clearly visible and labeled in the BFi waveform. (**d**–**e**) Significant brain activation and recovery to baseline can be seen in both the (**d**) $\Delta {\rm OD}={{\rm log}}_{10}{(I}_{0}/I(t))$ (p = 2.04 × 10^–7^) and (**e**) relative change in BFi (*p* = 1.06 × 10^–5^). Shown are the average $\Delta {\rm OD}$ and $\Delta$ BFi waveforms from fifteen trials. The green rectangular shaded region represents the time duration of a trial. The red shaded error region represents the standard error across the 15 trials.

Finally, in Fig. 7, we demonstrate experimentally the measurement of human CBF and brain activation at 33 mm source-detector separation using optimal parameter values (s/p = 0.84 and ${T}_{{\rm exp}}$ = 0.83 ms) for our system comprising the low-cost BAa camera and an 852 nm wavelength pulsed laser operating at PF = 10 as described in the Methods. Figure 7a shows the SCOS measurement setup which is described in detail in the Methods. We see in Fig. 7b, c the cardiac pulsatile signal in mean intensity $\langle I\rangle$ and BFi = 1/${K}_{{\rm f}}^{2}$, respectively, as measured using our SCOS system. No frame averaging was performed. The magnitude of the nonlinearity-induced ${K}^{2}$ error for the BAa camera (Fig. 1f) within the $\langle I\rangle$ range of the measurement (25–28 DN) is less than 4 × 10^–4^, or less than 2% of the average ${K}_{{\rm f}}^{2}$ (2.4 × 10^–2^) of the measurement. Morphological features of the cardiac signal including the three peaks (P1, P2, P3) and dicrotic notch are clearly visible in the BFi signal (Fig. 7c) while absent from the intensity signal (Fig. 7b). In Fig. 7d, e, we show measurement of brain activation in response to a mental subtraction task described in the Methods. To this end, we first used a commercial high-density fNIRS system to locate the activation region on the forehead with the largest task-averaged change in total hemoglobin concentration. We then placed our fiber-based SCOS source and detector optodes in the same region to measure changes in both optical density (OD) and BFi. We see that both ΔOD = ${{\rm log}}_{10}{(I}_{0}/I(t))$ (Fig. 7d) and ΔBFi (Fig. 7e) show significant (ΔOD: p = 2.04 × 10^–7^; ΔBFi: p = 1.06 × 10^–5^, two-tailed t-test) increases during brain activation and relaxation to baseline after activation.

## Discussion

We have shown how CMOS camera noise and nonidealities can significantly impact SCOS measurement SNR and accuracy in the low-photon-flux regime relevant to human brain measurements. As the information on specific camera characteristics needed to accurately correct for noise and to determine a camera’s suitability for SCOS is not available from published camera specifications, there is a need for a guide such as the one presented in this work to allow SCOS system developers to characterize, select, and optimize cameras on their own. By illustrating our guide using three cameras from different price ranges and showing that higher-end cameras designed for general imaging applications are not necessarily more suitable for SCOS than lower-end cameras, we have underscored the importance of approaching camera selection for SCOS based on the evaluation of specific camera attributes that are relevant to SCOS. Following our procedure to optimize the SCOS system, we achieved with the relatively inexpensive BAa CMOS camera (~ $500 at the time of purchase) comparable cardiac pulsatile waveform quality and recovery of brain activation (Fig. 7) to that of our previous human measurement with the HA scientific CMOS camera (~ $26,000 at the time of purchase)^26^. Note that when we were using the HA camera, we had to lower the laser power for subjects on multiple occasions, only to ensure that the system was operating in a more linear region of the photon transfer curve where the nonlinearity-induced $${K}^{2}$$ error was negligible compared to the average $${K}_{{\rm f}}^{2}$$ at the time (~ 3 × 10^–3^). This reduced the photon flux and hence the SNR.

Regarding camera nonlinearity, which is the first nonideality examined in our procedure, we note that published camera specifications typically provide a linearity metric only for the first-order response, defined as the mean intensity $\langle I\rangle$ versus the illumination power $\Phi$. While good first-order linearity alone might be enough for imaging applications that are primarily concerned with capturing intensity information, SCOS requires good linearity in the second-order response as well, defined as the variance of intensity ${\sigma }^{2}\left(I\right)$ versus the illumination power $\Phi$ (See Supplemental Fig. S1). Hence in our procedure we assess the linearity of the camera’s photon transfer curve (${\sigma }^{2}\left(I\right)$ vs. $\langle I\rangle$) which contains information about both the first-order and second-order responses. Since camera manufacturers do not typically publish photon transfer curves with sufficient resolution in lower camera counts, the user should follow the procedure presented in this work to evaluate the linearity of the photon transfer curve on their own, with particular attention paid to the intensity range that the user expects to utilize in actual SCOS measurements.

While we have corrected for the camera’s spatial nonuniformity in dark offset (also known as dark signal nonuniformity or DSNU) by subtracting the dark offset image from every raw image taken in a measurement, we do not explicitly correct for the impact of spatial nonuniformity in gain (also known as photoresponse nonuniformity or PRNU) on the estimation of the shot-noise-induced speckle contrast. Both types of nonuniformity arise from the implementation of individual amplifiers per pixel in CMOS cameras. In our procedure, we measure the photon transfer curve and estimate the average camera gain across all pixels. If the user intends to use sub-regions of the pixel array, we suggest measuring the photon transfer curve and estimating the gain within those sub-regions if significant nonuniformity in gain is suspected (we saw evidence of this in the HA camera). Note that measuring the photon transfer curve from less pixels may mean that more images would be needed to adequately suppress the noise in the mean intensity $\langle I\rangle$ and variance of intensity ${\sigma }^{2}\left(I\right)$ in the photon transfer curve.Another major camera parameter that a user is likely to consider when choosing between cameras is maximum frame rate. The required frame rate is largely dictated by the user’s application. For instance, a frame rate of at least 100 fps is preferred to sufficiently sample the pulsatile blood flow waveform for the extraction of hemodynamic parameters such as pulsatility index, critical closing pressure, and cerebrovascular resistance^33^. To achieve the above acquisition rate, DCS requires cardiac-gated averaging of as many as 50 arterial pulses to overcome the relatively low SNR, which distorts the pulsatile waveform and slows the measurement rate of hemodynamic parameters^33^. With SCOS, a user can potentially perform pulsatile waveform analysis with little to no cardiac-gated averaging, allowing for estimation of hemodynamic parameters at the cardiac frequency. We note that increasing the camera frame rate beyond the rate associated with the speckle decorrelation time—typically on the order of 10 μs for human CBF measurements—would not provide any SNR benefit from frame averaging because the speckle observations no longer become independent.

We decided to highlight camera nonlinearity, quantization distortion, and read noise variability among camera nonidealities because of their generalizability across cameras and impact on SCOS measurement accuracy. There are other less generalizable nonidealities that a user might encounter when testing different cameras. For example, with the BAa camera, we observed that at certain camera settings (i.e., > 16 dB analog gain value), it produced intermittent intensity jumps of 1–2 DN in magnitude that were independent of any changes in illumination (See supplementary Fig. S3). Since ${K}^{2}$ is a function of intensity, these intensity jumps would introduce random step-like biases in ${K}^{2}$ and distort the resulting BFi waveforms. We observed the same behavior across multiple BAa cameras, though not all of the ones we had access to. Our solution was to avoid the range of analog gain values (16–24 dB) where this instability occurred. As another example, we observed that the BAd camera in its available 12-bit depth mode skipped every 9^th^ DN value, which appeared as empty bins in an intensity histogram of the raw images. The skipped counts would distort the calculated ${K}^{2}$ at the intensities corresponding to the skipped counts, and thus we disregarded the BAd camera’s 12-bit mode and used its 8-bit mode which doesn’t exhibit this behavior. In general, any nonideality that can impact either $\langle I\rangle$ (e.g., first-order nonlinearity, dark offset instability, skipped counts) or $\sigma \left(I\right)$ (e.g., second-order nonlinearity, read noise instability) needs to be considered.

While we identified the BAa as the preferred camera for our SCOS system among the three cameras evaluated in this work, we note that the BAa camera may not necessarily be the optimal camera for other SCOS systems, since the overall performance of a SCOS system depends on the interplay between the camera and other system components such as the laser and the optics, such as choice of wavelength and fiber diameter, and because we have only evaluated a small sample of cameras among the pool of commercially available options. In addition, for our SNR simulations we assumed a fixed average photon flux per speckle of 22,619 s^-1^ equal to the value obtained during our mental subtraction experiment in Fig. 7. The photon flux per speckle can vary with the choice of source-detector separation, brain region measured, and subjects. We invite the user to utilize our recently developed noise model^25^, which was extended in this work to include the effects of the pixel-count-limited regime (Fig. 5) and pulsed illumination (Fig. 6), to predict the performance for a specific set of SCOS system parameters and experimental parameters. In conclusion, the camera characterization and parameter optimization procedure presented in this work serves as a guide for evaluating, optimizing, and comparing any set of cameras that a user wishes to consider for their SCOS system.

### Supplementary Information

Supplementary Information.

## Data Availability

The data that support the findings of this study are available from the corresponding author on reasonable request.

## Code availability

The code used to produce the results in this work were written in MATLAB and Python. The code is available from the corresponding author on reasonable request.

## References

[1] Bandera E (2006). Cerebral blood flow threshold of ischemic penumbra and infarct core in acute ischemic stroke. Stroke.

[2] Leigh R, Knutsson L, Zhou J, van Zijl PC (2018). Imaging the physiological evolution of the ischemic penumbra in acute ischemic stroke. J. Cereb. Blood Flow Metab..

[3] Aracki-Trenkic A (2020). ASL perfusion in acute ischemic stroke: The value of CBF in outcome prediction. Clin. Neurol. Neurosurg..

[4] Bouma GJ, Muizelaar JP (1992). Cerebral blood flow, cerebral blood volume, and cerebrovascular reactivity after severe head injury. J. Neurotrauma.

[5] Kelly DF (1997). Cerebral blood flow as a predictor of outcome following traumatic brain injury. J. Neurosurg..

[6] Golding EM, Robertson CS, Bryan RM (1999). The consequences of traumatic brain injury on cerebral blood flow and autoregulation: a review. Clin. Exp. Hypertens. N. Y. N.

[7] Korte N, Nortley R, Attwell D (2020). Cerebral blood flow decrease as an early pathological mechanism in Alzheimer’s disease. Acta Neuropathol. (Berl.).

[8] Cruz Hernández JC (2019). Neutrophil adhesion in brain capillaries reduces cortical blood flow and impairs memory function in Alzheimer’s disease mouse models. Nat. Neurosci..

[9] Wierenga CE, Hays CC, Zlatar ZZ (2014). Cerebral blood flow measured by arterial spin labeling MRI as a preclinical marker of Alzheimer’s disease. J. Alzheimers Dis. JAD.

[10] Cheng X, Sie EJ, Naufel S, Boas DA, Marsili F (2021). Measuring neuronal activity with diffuse correlation spectroscopy: a theoretical investigation. Neurophotonics.

[11] Durduran T (2004). Diffuse optical measurement of blood flow, blood oxygenation, and metabolism in a human brain during sensorimotor cortex activation. Opt. Lett..

[12] Jaillon F, Li J, Dietsche G, Elbert T, Gisler T (2007). Activity of the human visual cortex measured non-invasively by diffusing-wave spectroscopy. Opt. Express.

[13] Li J (2008). Transient functional blood flow change in the human brain measured noninvasively by diffusing-wave spectroscopy. Opt. Lett..

[14] Liu W (2021). Fast and sensitive diffuse correlation spectroscopy with highly parallelized single photon detection. APL Photonics.

[15] Bi R, Dong J, Lee K (2013). Deep tissue flowmetry based on diffuse speckle contrast analysis. Opt. Lett..

[16] Valdes CP (2014). Speckle contrast optical spectroscopy, a non-invasive, diffuse optical method for measuring microvascular blood flow in tissue. Biomed. Opt. Express.

[17] Liu J, Zhang H, Lu J, Ni X, Shen Z (2017). Quantitative model of diffuse speckle contrast analysis for flow measurement. J. Biomed. Opt..

[18] Lin C-HP (2023). Multi-mode fiber-based speckle contrast optical spectroscopy: analysis of speckle statistics. Opt. Lett..

[19] Robinson MB (2024). Comparing the performance potential of speckle contrast optical spectroscopy and diffuse correlation spectroscopy for cerebral blood flow monitoring using Monte Carlo simulations in realistic head geometries. Neurophotonics.

[20] Huang C (2016). Low-cost compact diffuse speckle contrast flowmeter using small laser diode and bare charge-coupled-device. J. Biomed. Opt..

[21] Dragojević T (2018). Compact, multi-exposure speckle contrast optical spectroscopy (SCOS) device for measuring deep tissue blood flow. Biomed. Opt. Express.

[22] Xu J, Jahromi AK, Brake J, Robinson JE, Yang C (2020). Interferometric speckle visibility spectroscopy (ISVS) for human cerebral blood flow monitoring. APL Photonics.

[23] Biswas, A., Mohammad, P. P. S., Moka, S., Takshi, A. & Parthasarathy, A. B. Non-invasive low-cost deep tissue blood flow measurement with integrated Diffuse Speckle Contrast Spectroscopy. *Front. Neuroergonomics* **4** (2024). 10.3389/fnrgo.2023.1288922 PMC10790947 38234484

[24] Favilla CG (2024). Validation of the Openwater wearable optical system: cerebral hemodynamic monitoring during a breath-hold maneuver. Neurophotonics.

[25] Zilpelwar S (2022). Model of dynamic speckle evolution for evaluating laser speckle contrast measurements of tissue dynamics. Biomed. Opt. Express.

[26] Kim B (2023). Measuring human cerebral blood flow and brain function with fiber-based speckle contrast optical spectroscopy system. Commun. Biol..

[27] Sun S, Hayes-Gill BR, He D, Zhu Y, Morgan SP (2015). Multi-exposure laser speckle contrast imaging using a high frame rate CMOS sensor with a field programmable gate array. Opt. Lett..

[28] Miao P, Lu H, Liu Q, Li Y, Tong S (2011). Laser speckle contrast imaging of cerebral blood flow in freely moving animals. J. Biomed. Opt..

[29] Zhao M, Huang C, Mazdeyasna S, Yu G (2021). Extraction of tissue optical property and blood flow from speckle contrast diffuse correlation tomography (scDCT) measurements. Biomed. Opt. Express.

[30] Labsphere. Technical Guide: Integrating Sphere Uniform Light Source Applications. (2008).

[31] Bennett WR (1948). Spectra of quantized signals. Bell Syst. Technol. J..

[32] Widrow, B. & Kollár, I. *Quantization Noise: Roundoff Error in Digital Computation, Signal Processing, Control, and Communications*. (Cambridge University Press, 2008).

[33] Wu KC (2023). Enhancing diffuse correlation spectroscopy pulsatile cerebral blood flow signal with near-infrared spectroscopy photoplethysmography. Neurophotonics.

