# Supplementary material for: Choosing a camera and optimizing system parameters for speckle contrast optical spectroscopy
Source: Sci Rep. 2024 May 24;14:11915. doi: 10.1038/s41598-024-62106-y (PMC11126420; doi:10.1038/s41598-024-62106-y)
Supplement: Supplementary file 1 — Supplementary Information. [file 41598_2024_62106_MOESM1_ESM.docx]

**Supplement to Choosing a camera and optimizing system parameters for speckle contrast optical spectroscopy**

**Tom Y. Cheng^1,2,3^***^+^***, Byungchan Kim^1^***^+^***, Bernhard B. Zimmermann^1^, Mitchell B. Robinson^2^, Marco Renna^2^, Stefan A. Carp^2^, Maria Angela Franceschini^2^, David A. Boas^1^, Xiaojun Cheng^1,*^**

*^1^Neurophotonics Center, Department of Biomedical Engineering, Boston University, Boston, MA 02215, USA*

*^2^Athinoula A. Martinos Center for Biomedical Imaging, Department of Radiology, Massachusetts General Hospital, Harvard Medical School, Charlestown, MA 02129, USA*

*^3^Lincoln Laboratory, Massachusetts Institute of Technology, Lexington, MA 02421, USA*

*^+^These authors contributed equally*

[*^*^xcheng17@bu.edu*](mailto:*xcheng17@bu.edu)

^
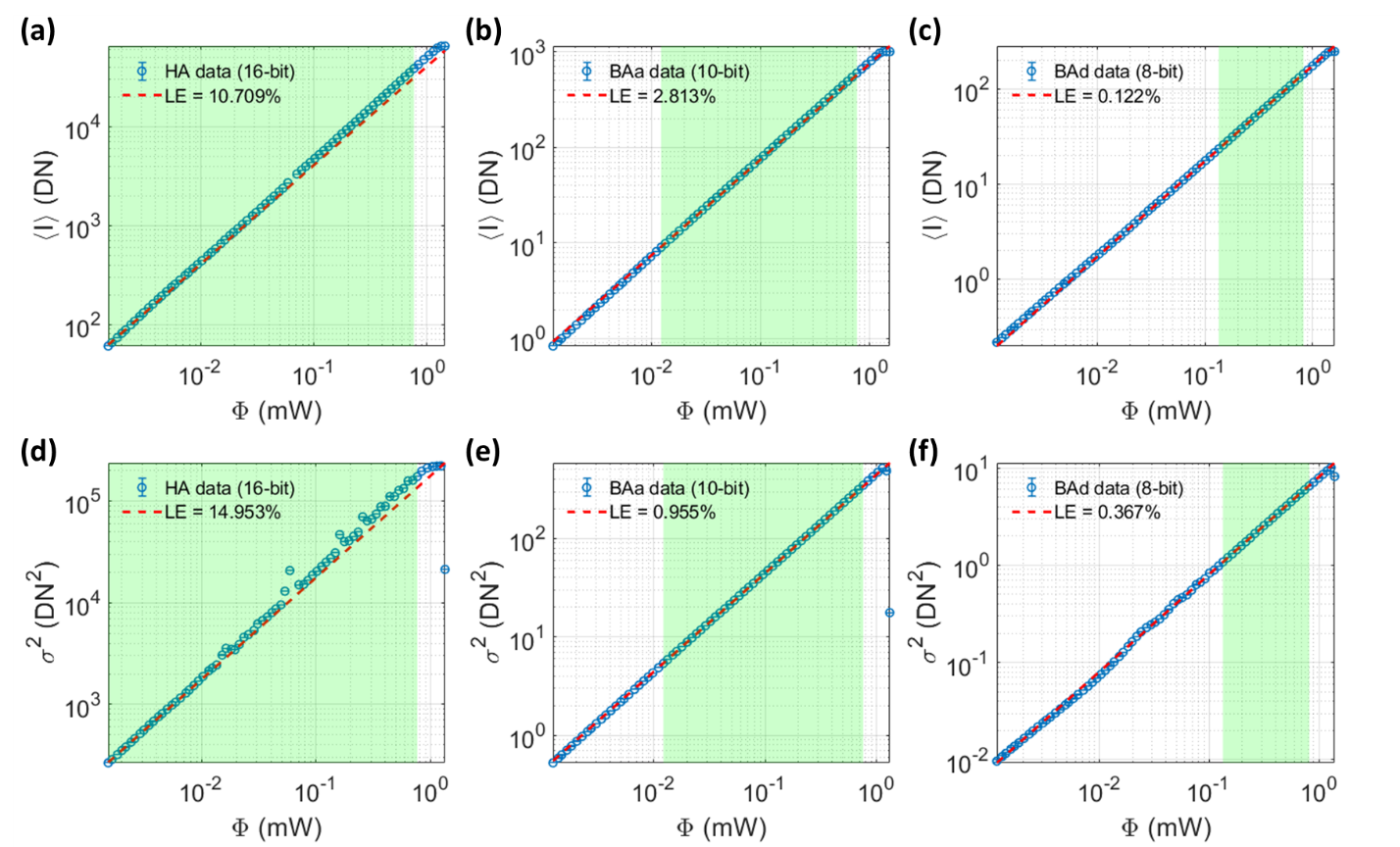
^

**Figure S1. Characterization of first-order and second-order linearity for the three CMOS cameras.** (a)-(c) First-order linearity for the three cameras (HA, BAa, BAd), measured as the mean intensity $\left\langle I \right\rangle$ versus the measured optical power $\Phi$ of the LED illumination. Here the optical power of the LED is used as a proxy for the mean number of incident photons on the sensor. Blue circles are the experimental data and red dashed lines are the linear regression lines. Error bars represent the temporal standard deviation of the mean of each image obtained. The linear regression is performed on data within a limited $\Phi$ range as indicated by the shaded region, where the shot noise is greater than twice the read noise and the camera is at less than 70% of its saturation intensity. The linearity error (LE) is calculated as the mean magnitude of the relative deviation of the measured variance from the regression line within the fitting range. (d)-(f) Second-order linearity for the three cameras, defined as the variance of the intensity $\sigma^{2}$ versus the mean intensity $\left\langle I \right\rangle$. The read noise variance offset has been subtracted from all data points. Error bars represent the temporal standard deviation of the variance of each difference image obtained. HA: Hamamatsu Orca Fusion BT C15440-20UP; BAa: Basler a2A1920-160umPRO; BAd: Basler daA1280-54um.

1. **Characterization of first-order and second-order linearity for the three CMOS cameras**

Fig. S1 shows both the first-order linearity and the second-order linearity for each of the three cameras evaluated. While good first-order linearity alone may be sufficient for imaging applications primarily concerned with representation of intensity information, in SCOS, where the standard deviation of the intensity $\sigma\left( I \right)$ is used in addition to the mean intensity $\left\langle I \right\rangle$, well-behaved second-order linearity is also required. Deviations in both the $\sigma\left( I \right)$ and $\left\langle I \right\rangle$ from linear behavior contribute to nonlinearity in the photon transfer curve ($\sigma^{2}\left( I \right)$ vs. $\left\langle I \right\rangle$) and systematic error in the contrast $K$. The magnitude of the systematic error in $K^{2}$ due to the systematic nonlinearities is given by $\left| \Delta K^{2} \right|=\left| \left[ \sigma^{2}\left( I \right)-\sigma_{fit}^{2}\left( I \right) \right]/\left\langle I \right\rangle^{2} \right|$, where $\sigma_{fit}^{2}\left( I \right)$ is the variance of the intensity predicted from the linear fit to the photon transfer curve. Nonlinearity of the first-order and second-order responses at lower $\Phi$ values causes the linear fits to diverge from the data at higher $\Phi$ values, as can be seen most clearly for the HA camera (Fig. S1a, S1d).

**
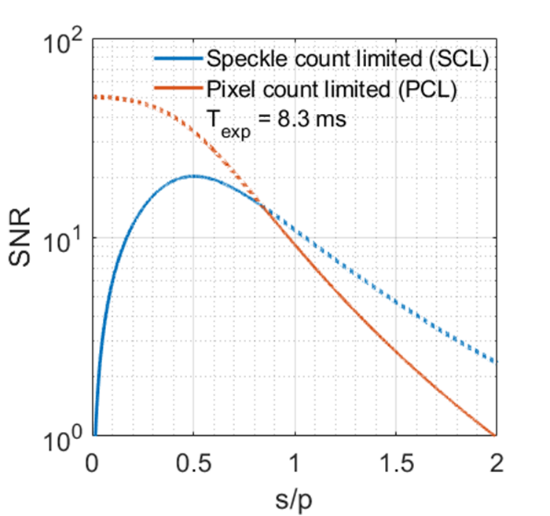
**

**Figure S2. SCOS SNR dependence on s/p ratio for a camera with higher read noise.** SNR versus s/p at $T_{exp}$ = 8.3 ms for speckle-count-limited and pixel-count-limited cases. All parameters are identical to those used in Fig. 5 except for the camera’s RMS read noise, which has been artificially increased in simulation from 1.97 e^-^ to 10 e^-^. When the system is speckle-count-limited, the total number of speckles/fiber modes is fixed at M = $3.3\times{10}^{6}$, estimated from the total area of the fiber output and the s/p ratio obtained experimentally. In the speckle-count-limited case, we assume that the camera has enough pixels to image all the speckles. When the system is pixel-count-limited, the total number of pixels is fixed at 1936×1216 pixels for the BAa camera, and we assume all pixels are filled with speckles.

1. **SCOS SNR dependence on s/p ratio for a camera with higher read noise**

Fig. S2 shows the SNR dependence on s/p ratio for a hypothetical camera with identical parameters to that of the BAa camera used in Fig. 5 except for the read noise, which has been increased in simulation from 1.97 e^-^ to 10 e^-^. A read noise value of 10 e^-^ is within the typical range among commercial CMOS cameras. The fiber-based SCOS system’s SNR as a function of s/p follows the solid red and blue lines in Fig. S2. In contrast to the scenario in Fig. 5b, we see in Fig. S2 that the maximum SNR is achieved not at the point of intersection of the two SNR curves where the fiber’s output image is matched to the size of the camera’s pixel array, but at a reduced s/p value of 0.5 where the fiber’s output image is smaller than the camera’s pixel array. Reducing the s/p value increases the photon flux per pixel and pushes the camera into the shot-noise-limited regime. The resulting benefit to SNR as s/p is decreased overcomes the SNR reduction from decreased spatial sampling of the speckle pattern, until s/p is reduced to 0.5.


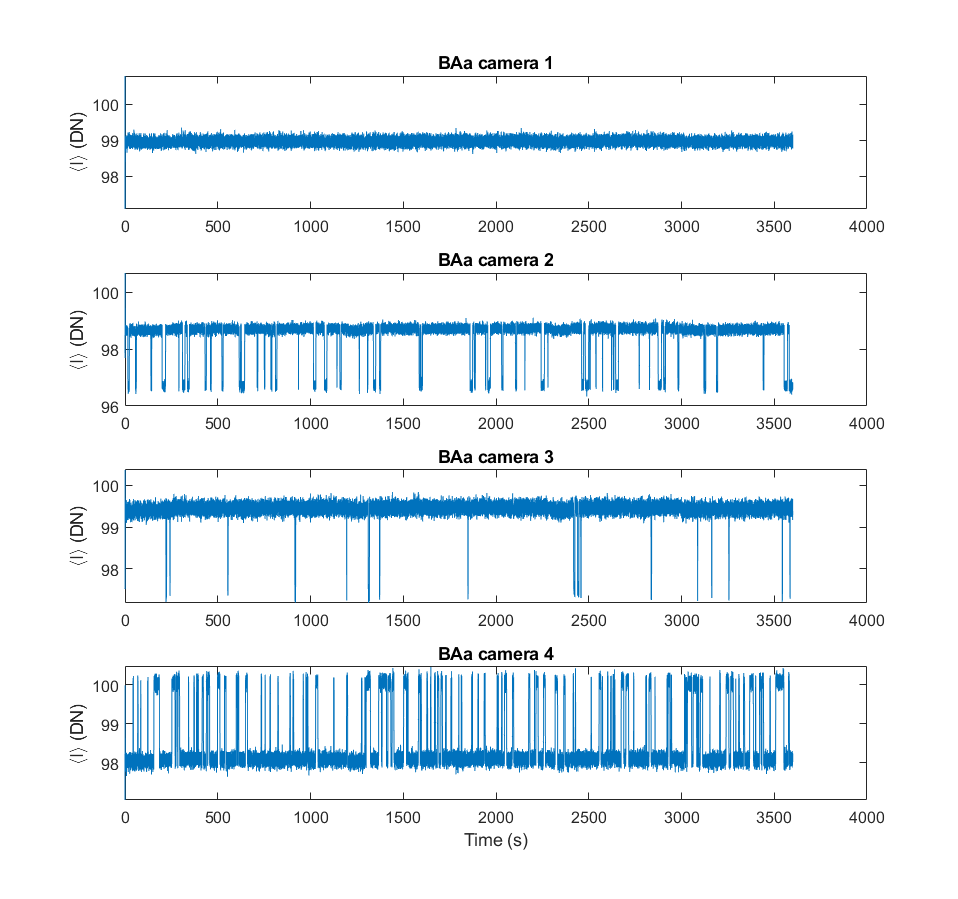


**Figure S3. Intermittent jumps in mean camera intensity from different BAa cameras at a high analog gain setting.** Shown is the mean camera intensity $\left\langle I \right\rangle$ versus time in seconds for four different BAa cameras with identical camera settings (12-bit depth and 24 dB analog gain). Three out of the four cameras show random jumps in $\left\langle I \right\rangle$ and the three cameras exhibit different recurrence rates in the jumps, which suggest that this is a camera-specific phenomenon. All cameras acquired images in the dark.
